# Targeting *N*-glycosylation of 4F2hc mediated by glycosyltransferase B3GNT3 sensitizes ferroptosis of pancreatic ductal adenocarcinoma

**DOI:** 10.1038/s41418-023-01188-z

**Published:** 2023-07-21

**Authors:** Heng Ma, Xianlong Chen, Shengwei Mo, Yue Zhang, Xinxin Mao, Jingci Chen, Yilin Liu, Wei-Min Tong, Zhaohui Lu, Shuangni Yu, Jie Chen

**Affiliations:** 1 Department of Pathology, Peking Union Medical College Hospital, Peking Union Medical College and Chinese Academy of Medical Science, Beijing, 100730 China; 2 Department of Pathology, Institute of Basic Medical Sciences, Peking Union Medical College and Chinese Academy of Medical Science, Beijing, 100730 China

**Keywords:** Glycoproteins, Gastroenteritis

## Abstract

Pancreatic ductal adenocarcinoma (PDAC) remains a highly fatal malignancy partially due to the acquired alterations related to aberrant protein glycosylation that pathologically remodel molecular biological processes and protect PDAC cells from death. Ferroptosis driven by lethal lipid peroxidation provides a targetable vulnerability for PDAC. However, the crosstalk between glycosylation and ferroptosis remains unclear. Here, we identified 4F2hc, a subunit of the glutamate-cystine antiporter system X_c_^–^, and its asparagine (*N*)-glycosylation is involved in PDAC ferroptosis by *N*- and *O*-linked glycoproteomics. Knockdown of *SLC3A2* (gene name of 4F2hc) or blocking the *N*-glycosylation of 4F2hc potentiates ferroptosis sensitization of PDAC cells by impairing the activity of system X_c_^–^ manifested by a marked decrease in intracellular glutathione. Mechanistically, we found that the glycosyltransferase B3GNT3 catalyzes the glycosylation of 4F2hc, stabilizes the 4F2hc protein, and enhances the interaction between 4F2hc and xCT. Knockout of *B3GNT3* or deletion of enzymatically active B3GNT3 sensitizes PDAC cells to ferroptosis. Reconstitution of 4F2hc-deficient cells with wildtype 4F2hc restores ferroptosis resistance while glycosylation-mutated 4F2hc does not. Additionally, upon combination with a ferroptosis inducer, treatment with the classical *N*-glycosylation inhibitor tunicamycin (TM) markedly triggers the overactivation of lipid peroxidation and enhances the sensitivity of PDAC cells to ferroptosis. Notably, we confirmed that genetic perturbation of *SLC3A2* or combination treatment with TM significantly augments ferroptosis-induced inhibition of orthotopic PDAC. Clinically, high expression of 4F2hc and B3GNT3 contributes to the progression and poor survival of PDAC patients. Collectively, our findings reveal a previously unappreciated function of *N*-glycosylation of 4F2hc in ferroptosis and suggest that dual targeting the vulnerabilities of *N*-glycosylation and ferroptosis may be an innovative therapeutic strategy for PDAC.

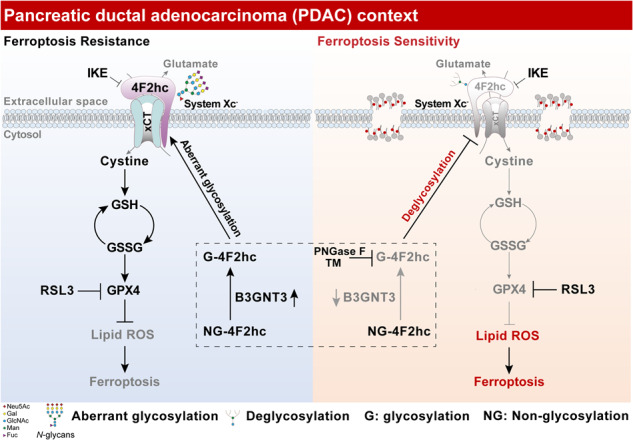

## Introduction

Ferroptosis is a novel form of regulated cell death characterized by the iron-dependent unrestricted toxic accumulation of lipid peroxidation products and plasma membrane rupture and was termed by Dixon et al. in 2012 [1]. Research in this field has revealed various targetable vulnerabilities in cell metabolism, redox homeostasis, and iron handling, which may provide potential intervention targets for anticancer therapy [2, 3]. Among the numerous regulatory pathways involved in ferroptosis defense, the glutamate-cystine antiporter system X_c_^–^ is widely known and consists of two subunits, namely, the heavy chain subunit 4F2hc (also known as CD98hc, encoded by the SLC3A2 gene) and the light chain subunit xCT (encoded by the SLC7A11 gene), which mediate the synthesis of cysteine-derived antioxidants [4]. *SLC7A11* has been identified as the target gene of nuclear factor erythroid 2-related factor 2 (NRF2), which is responsible for maintaining redox homeostasis during oxidative stress [5, 6]. xCT predominantly functions in the intracellular transfer of extracellular cystine, and xCT seems to be more critical than 4F2hc in ferroptosis regulation. However, we noticed that 4F2hc, a glycoprotein, is extremely important for maintaining xCT protein stability and appropriate membrane localization. In terms of structural biology, 4F2hc controls the intracellular trafficking and membrane topology of its heterodimerization partner [7] through polar and hydrophobic interactions. For example, residues in 4F2hc form a short helix to fix xCT on the intracellular side [8]. Thus, 4F2hc is required for the recruitment of xCT to the plasma membrane, its degradation ultimately results in xCT functional inactivation [9, 10]. In addition, recent studies have shown that deletion of 4F2hc causes the decompensation of system X_c_^–^, indicating that 4F2hc plays an equally important role in inhibiting ferroptosis [11–13]. However, as a membrane glycoprotein, 4F2hc has not been extensively studied in the context of ferroptosis.

Disease-associated stress may pathologically remodel the proteome or cause protein connectivity dysfunction, especially molecular chaperones [14]. Glycosylation, a widespread posttranslational modification (PTM) [15], is a finely tuned enzymatic reaction process that occurs mainly in the endoplasmic reticulum (ER) and Golgi apparatus, where glycosyltransferases and glycosidases add glycans to proteins and lipids. There are two types of glycosylation: *N*-glycosylation and *O*-glycosylation [16]. Glycosylation of proteins stabilizes proteins, helps proteins move to the right location, and guides molecular chaperones to fold properly. Additionally, emerging evidence indicates that aberrant protein glycosylation plays a critical role in cell death evasion, sustained proliferative signaling, and chemoresistance in various malignancies, including pancreatic ductal adenocarcinoma (PDAC) [17, 18]. Both glycosylation and ferroptosis are physiological metabolic processes that may provide new therapeutic opportunities for cancer treatment. However, hitherto the crosstalk between glycosylation and ferroptosis has not yet been largely revealed.

PDAC accounts for more than 90% of pancreatic cancer cases and is often life-threatening, with a 5-year relative survival rate of approximately 11%. According to its higher incidence and mortality rates, PDAC is projected to rank as the second leading cause of cancer-related death by 2040 [19, 20]. Accumulating evidence indicates that targeting ferroptosis could exploit a vulnerability in cancer, especially PDAC, which is closely intertwined with *KRAS* mutant-driven activation of the antioxidant system and is rich in iron [21]. Previous preclinical studies have indicated that suppressing system X_c_^–^ in the extrinsic pathway (such as system X_c_^–^ inhibition and cysteine depletion [22]) or directly reducing the activity or expression of the core antioxidant molecule in the intrinsic pathway (such as inhibitors targeted for GPX4 [23], FSP1 [24] and DHODH [25]) might effectively render PDAC cells susceptible to ferroptosis. But the success of such therapeutic strategies remains limited because of ferroptosis resistance and some other unknown underlying mechanisms [26]. Therefore, a deeper understanding of ferroptosis resistance may reveal new ferroptosis-related mechanisms and provide more optimized treatment options for PDAC.

In this study, we performed integrative *N*- and *O*-linked glycoproteomics and functional analyses to reveal a previously unrecognized coupling between PDAC ferroptosis and *N*-glycosylation of 4F2hc, and demonstrate that disturbs the process of 4F2hc glycosylation can induce susceptibility of PDAC to ferroptosis.

## Materials and methods

### Patient specimens and tissue microarray (TMA) construction

A total of 291 patients diagnosed with primary PDAC between January 2015 and July 2019 were consecutively recruited from the pathology archives of the Peking Union Medical College Hospital (PUMCH) (Beijing, China). Patients who had received neoadjuvant therapy and who died owing to postoperative complications or lacked follow-up information were excluded from this retrospective study. Hematoxylin and eosin (HE)-stained slides from all patients were retrieved and reviewed by two pathologists (ZL and SY) who were blinded to the patient’s clinical outcomes. In cases of disagreement, a third pathologist (JC) confirmed the histological diagnosis. Detailed clinicopathological data and prognostic information of all patients were collected from the medical records and telephone interviews. The TMA with a single 2-mm core per case was constructed using a Manual Tissue Microarrayer (MiniCore, Mitogen, Hertford, UK). Briefly, representative tumor areas were marked on a HE stained slide and then punched from each donor formalin-fixed paraffin-embedded (FFPE) block for the recipient TMA blocks.

### Cell lines and reagents

Human cell lines HPNE, PANC-1, MIA PaCa-2, BxPC-3, and AsPC-1 were obtained from the National Biomedical Cell Resource Center (Beijing, China) and identified by STR Profiling (D2081-2084) as well as tested negative for contamination. Cells were maintained at 37 °C in 5% CO_2_ incubator. HPNE were grown in 75% Dulbecco’s modified Eagle’s medium (DMEM) without glucose (Sigma, D5030-10 × 1 L; St. Louis, MO, USA) and 25% Medium M3 Base (Incell corp, M300F-500; Texas, USA), supplemented with 1% penicillin and streptomycin (Gibco, 1514022; Massachusetts, USA), 10% fetal bovine serum (FBS) (Corning, 35-010-CV; New York, USA), 5.5 mM D-glucose (Sigma-Aldrich, G7021-100G), 10 ng/ml human recombinant EGF (PeProtech, AF-100-15; New Jersey, USA), 1 × GlutaMax^TM^ Supplement (Gibco, 35050061), and 750 ng/ml puromycin(Gibco, A1113803). The remaining cancer cells were maintained in DMEM (Corning, 10-013-CV) or RPMI 1640 (Corning, 10-040-CV) supplemented with 10% FBS. All cell lines were cultured in 6–10 cm dishes or plated in 6–24 well plates for the indicated experiment. All chemical compounds used in cell culture experiments included RSL3 (Selleck, S8155; Houston, USA), erastin (Selleck, S7242), imidazole ketone erastin (Selleck, S8877), Liproxstatin-1 (Selleck, S7699), ±-α-tocopherol (Selleck, S6104), UAMC-3203 (Selleck, S8792), Z-VAD-FMK (Selleck, S7023), Necrostatin-1 (Selleck, S8037), cycloheximide (Selleck, S7418), tunicamycin, and protein glycosylation inhibitor (Abcam, ab120296).

### LC-MS/MS for glycoproteomics analysis

1 × 10^6^ PANC-1 cells were plated in 75 cm^2^ cell culture flasks (Corning, 430640, New York, USA) until they reached 90% cell confluence and then incubated with the indicated concentration of RSL3 or not for 12 h (*n* = 3 per group). Following treatment, cells were lysed using 300 μL 8 M urea with 1% protease inhibitor and centrifuged at 14,000 × g for 20 min at 4 °C, and the supernatant was collected. The protein concentrations of the lysates were determined using the Bradford methods (Thermo Scientific, Pierce^TM^ BCA protein assay kit, 23225, Massachusetts, USA). The details are provided in the “Supplementary Materials and Methods”.

### Cycloheximide chase analysis

To evaluate the stability of the 4F2hc protein (including but not limited to), cycloheximide (CHX) (20 μM) was added to indicate cells that had been treated with tunicamycin or transfected with sg*B3GNT3* for 0, 2, 4, and 8 h. Then collection each sample was collected and subjected to western blotting, and the relative intensities of 4F2hc protein were quantified using Image J software.

### Enzymatic deglycosylation analysis of interested glycoprotein

To validate the glycosylation of 4F2hc proteins, PNGase F (New England BioLabs, P0704S, USA) and *O*-glycosidase (New England BioLabs, P0733, USA) were used according to the manufacturer’s protocol. Briefly, 20 μg protein from the whole-cell lysates was added to a 10 μL total reaction volume comprising 1 μL of 10 × glycoprotein denaturing buffer and 9 μL water and then subjected to denaturation by heating at 100 °C for 10 min. After cooling to room temperature, 2 μL of NP-40, 2 μL of 10 × GlycoBuffer 2, and water were combined to make up a 20 μL reaction volume. Following incubation with or without 2 μL PNGase F at 37 °C overnight, the mixture was subjected to immunoblotting analysis with the 4F2hc antibody.

### RNA sequencing

Total RNA was extracted from PANC-1 cells treated with or without RSL3 (n = 3 per group) using the TRIzol reagent (Invitrogen, 15596018, Carlsbad, CA, USA). The purity and concentration of RNA were determined using a NanoDrop spectrophotometer (Thermo Scientific, Massachusetts, USA), and integrity was determined using an Agilent 2100 bioanalyzer (RNA 6000 Nano kit 5067-1511). The details are provided in the “Supplementary Materials and Methods”.

### Ferroptosis-related genes (FRGs) and glycosyltransferase genes

FRGs were retrieved from the FerrDb database (http://www.zhounan.org/ferrdb) [27], which included 255 drivers, 208 suppressors, and 125 markers (Fig. S1A and Table S1). After removing repetitive genes, 259 FRGs were eventually obtained and annotated into corresponding protein names for subsequent intersection with differential glycoprotein. A total of 207 known glycosyltransferase genes were collected from the RNA-Seq data for TCGA pancreatic cancer subjects [28], which were used to identify hub gene sets by intersecting with differentially expressed genes screened out from RNA sequencing for further analysis.

### Multiplex immunohistochemistry/immunofluorescence (mIHC/IF)

mIHC/IF staining was performed on TMA slides using an Opal Multiplex IHC Assay Kit (Akoya Biosciences, MA, USA), according to the manufacturer’s protocol. The details are provided in the “Supplementary Materials and Methods”.

### Flow cytometric detection of cell death and lipid ROS

1 × 10^5^ cells were plated in 6-well plates and incubated overnight, then treated with various compounds for 12 h. After treatment, for cell death analysis, viable cells and floating cells were collected and stained with 5 μL FITC Annexin V and 5 μL propidium iodide (BD Bioscience, Apoptosis Detection Kit I, 556547) 15 min. For lipid ROS detection, 5 μM of C11-BODIPY 581/591 (Invitrogen, D3861) was added and incubated with cells for 30 min at 37 °C, 5% CO2 in an incubator. Cells for both cell death analysis and lipid ROS detection were harvested and resuspended in 200 μL PBS, and then strained through a 40 μm cell strainer (Corning Falcon, 352350) for flow cytometric analysis (BD LSRFortessa). A minimum of 10,000 cells per well were analyzed in the FITC channel or in combination with the PI channel. The FlowJo v10 software was used to analyze cell death and lipid peroxidation.

### Animal experiments

Female and male BALB/c nude mice (4 weeks old) were purchased from the VitalRiver Laboratory Animal Technology Co., Ltd. (Beijing, China), and housed in the Center for Experimental Animals Research at the Institute of Basic Medical Science, Chinese Academy of Medical Science (CAMS). For the subcutaneous transplant tumor models, 2 × 10^6^ of *Cas9*^Control^ and *B3GNT3*^KO^ PANC-1 cells were suspended in 100 μL PBS and injected subcutaneously into the right side of immuno-deficient nude mice. Tumor growth was monitored using a Vernier caliper taken once per week for 28 days. For the orthotopic transplantation tumor models, 2 ×10^6^ of *Cas9*^Control^ and *B3GNT3*^KO^ PANC-1 cells, and 2 ×10^6^ of shControl and sh*SLC3A2* PANC-1 cells which stably expressed firefly luciferase were resuspended in PBS separately and were carefully injected into the surgically exposed pancreatic tail on day 7, and the pancreatic peritoneal cavity abdominal wall and skin were stitched. Tumor progression (shControl and sh*SLC3A2* groups) was monitored by bioluminescent imaging (IVIS Lumina III). To investigate the therapeutic effect of IKE (40 mg/kg) and its combination with tunicamycin (1 mg/kg) in nude mice bearing sh*SLC3A2* PANC-1 cells xenografts, the mice were treated with the above drugs every other day. The mice’s survival and tumor growth were also recorded. All tumors were surgically removed and processed for IHC on day 28. The tumor volume was calculated using the following formula: tumor volume = 1/2 × tumor length × tumor width^2^.

### Statistics and reproducibility

All statistical analyses were performed using the SPSS version. 22 or GraphPad Prism version 9. To compare two experimental conditions, the Mann-Whitney test or Student’s t-test was performed for unpaired samples, and the Wilcoxon rank-sum test was applied for all paired t-test. A two-way analysis of variance (ANOVA) was performed to compare the multiple experimental groups. Two-sided Pearson’s test or Spearman rank correlation test was used to identify correlations among the variables. The log-rank test and univariate and multivariate Cox regression analysis were performed to estimate the survival and determine the independent prognostic factors, respectively. No statistical methods were used to predetermine the sample size. Each experiment was repeated at least three times unless otherwise indicated in the figure legend. All data are presented as the mean ± SD unless otherwise stated. Statistical significance was set at a threshold of *P* value less than 0.05.

## Results

### *N*-/*O*-glycoproteomics and RNA-seq link *N*-glycosylated 4F2hc and glycosyltransferase B3GNT3 to PDAC ferroptosis

**Fig. 1:**
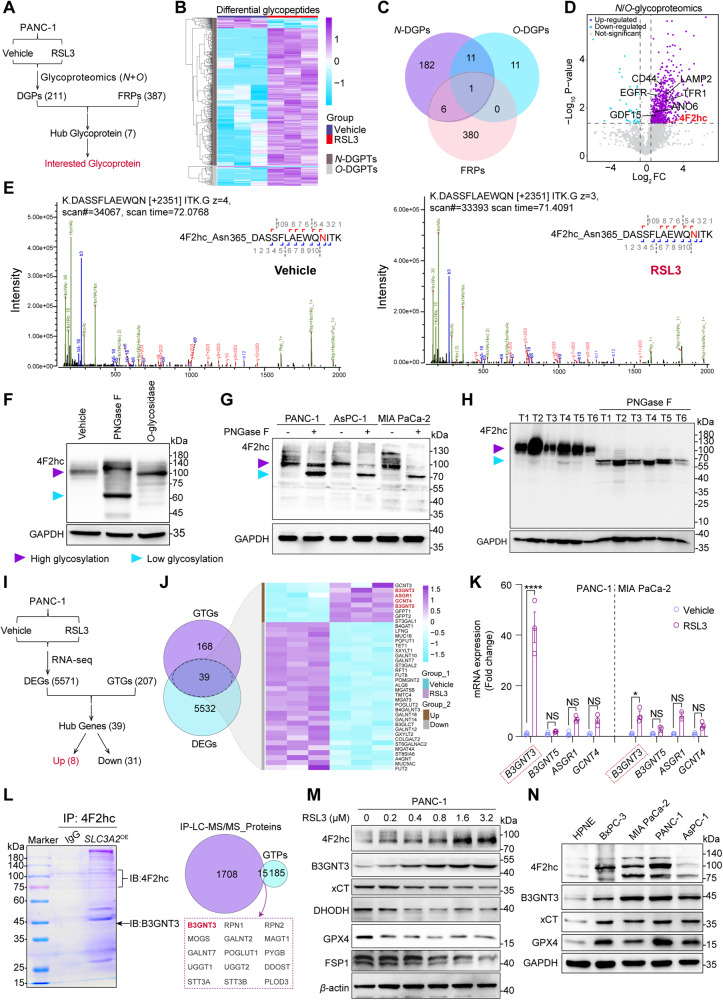
Integrated *N* + *O*-glycoproteomic and RNA-seq revealed that *N*-glycosylated 4F2hc and glycosyltransferase B3GNT3 were differentially upregulated during PDAC cell ferroptosis. **A**. Pipeline for identifying glycoproteins of interest shared by 211 DGPs and 387 ferroptosis-related proteins (FRPs). **B** Heatmap of normalized expression of differential glycopeptide across the vehicle and RSL3 groups. The upper and bottom panels show the differential *N*- and *O* -glycopeptides, respectively. The dark blue- and red bars denote vehicle and RSL3, respectively. The brown- and gray bars represent *N*-linked DGPs (*N*-DGPs) and *O*-linked DGPs (*O*-DGPs), respectively. **C** Venn diagram displaying the number of overlapping and unique proteins from 200 *N*-DGPs, 23 *O*-DGPs, and 387 FRPs. **D** Volcano plot showing 7 DGPs identified from *N-* and *O*-glycoproteomic. **E** LC–MS/MS-based analysis of the targeted differential *N*-linked glycosylation sites Asn365 of the 4F2hc in PANC-1 cells treated with or without 0.8 μM RSL3 for 12 h. The b11 and y4 ions represent the peptide fragmentation of Asn365 which was detected in RSL3-treated PANC-1 cells. **F** Western blotting analysis of the glycosylation changes in 4F2hc protein extracted from PANC-1 cells was treated with PNGase F and *O*-glycosidase for 1 h at 37 °C. **G** PANC-1, AsPC-1, and MIA PaCa-2 cells were treated with or without PNGase F for 1 h at 37 °C followed by immunoblot analysis. Purple- and cyan-triangle denote high- and low-glycosylation, respectively. **H** Cell lysates from six PDAC tumors treated with or without PNGase F for 1 h at 37 °C. **I** Flow chart presenting an investigation to screen out the putative target genes from glycosyltransferase genes (GTGs) and differentially expressed genes (DEGs) based on RNA-seq. **J** Left, Venn diagram displaying the number of overlapping genes from 207 GTGs and 5571 DEGs. Right, heatmap of all the up- and down-regulated hub genes intersection from 207 GTGs and 5571 DEGs across the vehicle and RSL3 groups. The brown bar represents upregulated DEGs, and the gray bar represents downregulated DEGs, the genes marked in red are four GTGs associated with PDAC prognosis. **K** qRT-PCR profiling of the expression of 4 selected upregulated genes involved in glycosyltransferases in PANC-1 and MIA PaCa-2 cells treated with vehicle or RSL3. **L** Coomassie blue stained SDS gels of affinity-purified protein complexes co-immunoprecipitated by the 4F2hc antibody from *SLC3A2* overexpressed PANC-1 cells (line 3). Venn diagram displaying the 15 overlapping proteins from 1708 precipitated proteins and 185 glycosyltransferases (GTPs). **M** Expression of 4F2hc, xCT (CST#12691, 35 kDa), B3GNT3, DHODH, GPX4, and FSP1 in PANC-1 cells treated with RSL3 at the range of concentrations for 12 h. **N** Western blot analyzing 4F2hc, xCT (Abcam#175186, 55 kDa), B3GNT3, and GPX4 in human pancreatic epithelial nestin-expressing (HPNE) and PDAC cells (BxPC-3, MIA PaCa-2, PANC-1, AsPC-1). The western blot experiment was representative of two biological replicates with similar results. All data are shown as the mean ± SD. Statistical significance among the indicated groups was assessed by ANOVA analysis or unpaired Student’s t-test.

Aberrant glycosylation of the proteome protects pancreatic ductal adenocarcinoma (PDAC) from cell death induction [29]. However, its role in PDAC ferroptosis has not been well studied. To this end, *N*-/*O*-glycoproteomics was performed to reveal the glycoproteomic characteristic of PDAC cells undergoing RSL3-induced ferroptosis (Fig. 1A). We first confirmed that most of the *N*- and *O*-linked differentially expressed glycopeptides (DGPTs), including 530 (86.74%) *N*-DGPTs and 37 (6.06%) *O*-DGPTs, were upregulated in PANC-1 cells treated with RSL3 (Fig. 1B, S1B). Accordantly, 191 (77.64%) *N*-linked differentially expressed glycoproteins (*N*-DGPs) and 21 (8.54%) *O*-linked DGPs were upregulated (Fig. S1C). Enrichment analyses of GeneOntology (GO) terms indicated that those upregulated DGPs were enriched in transport, signal transduction, and cell death (Fig. S1D, F). Additionally, the Kyoto Encyclopedia of Genes and Genomes (KEGG) pathway analysis of the upregulated *N*-DGPs highlighted the pathway in cancer (Fig. S1E, G). Finally, seven candidate DGPs were identified including 4F2hc, GDF15, ANO6, TFR1, LAMP2, EGFR, and CD44 after the intersection of 211 *N* + *O* DGPs and 387 ferroptosis-related proteins (FRPs) (Fig. 1C, D).

Remarkably, 4F2hc has drawn our attention, as it is widely reported to be critical for ferroptosis defense but the role of 4F2hc glycosylation in ferroptosis is poorly understood. To decipher the specific differential modification sites of 4F2hc affected by RSL3-induced ferroptosis, the tryptic glycopeptides of 4F2hc were analyzed by nanoscale LC–MS/MS. The results showed that Asn365 was identified as a unique differential upregulated *N*-glycosylation site of 4F2hc (Fold change = 6.09; *P*-value = 0.02), and the intensity of Asn365-specific glycoforms increased dynamically after ferroptosis activation (Fig. 1E). 4F2hc have four *N*-glycosylation sites include Asn365 predicted by NetNGlyc-1.0 (Fig. S1H). We confirmed that the *N*-glycosylation of endogenous 4F2hc was completely inhibited and the molecular weight of 4F2hc was reduced from ~100 to ~70 kDa when PANC-1 cells lysates were treated with peptide-*N*-glycosidase F (PNGase F), which is a recombinant glycosidase was used to remove asparagine-linked oligosaccharides from polypeptides, but not with recombinant *O*-glycosidase (Fig. 1F). Similar results were further confirmed in PANC-1, AsPC-1, and MIA PaCa-2 cells and six human PDAC tissues when treated with PNGase F (Fig. 1G, H). Together, these results indicated that 4F2hc is a highly *N*-glycosylated protein in PDAC and its *N*-glycosylation might play a potential role in ferroptosis execution.

To identify which glycosyltransferases are critical for glycosylation initiation during ferroptosis execution, 207 glycosyltransferase genes (GTGs) were collected from the reported literature. Parallel RNA sequencing (RNA-seq) was performed in PANC-1 cells treated with RSL3 or not. We identified approximately 5571 differentially expressed genes (DEGs) (Fig. 1I). Then, 39 hub genes were identified after the intersection of the DEGs and GTGs, including 8 upregulated genes and 31 downregulated genes (Fig. 1I, J). KEGG enrichment analysis demonstrated that ferroptosis was ranked among the top 10 pathways based on 2603 upregulated DEGs (Fig. S1I). Further analysis of 8 upregulated genes according to their mRNA expression from the TCGA datasets revealed that just B3GNT3, B3GNT5, and ASGR1 were highly expressed in PDAC tissues compared to normal samples except for GCNT4 (Fig. S2A–D). High gene expression levels of B3GNT3 and B3GNT5 were associated with a poor prognosis for patients with PDAC, but ASGR1 and GCNT4 showed the opposite pattern (Fig. S2E–H). The qRT-PCR validation experiment showed that only B3GNT3 was elevated considerably in indicated cells treated with RSL3 (Fig. 1K). Importantly, we identified B3GNT3, but not other glucosyltransferases, as a bona fide binding partner of 4F2hc protein in *SLC3A2*^OE^ PANC-1 cells analyzed by immunoprecipitation coupled with liquid chromatography-mass spectrometry/mass spectrometry (IP–LC–MS/MS) (Fig. 1L, Fig. S1J). Accordantly, 4F2hc was also identified in *B3GNT3*^OE^ PANC-1 cells (Fig. S1K). Co-immunoprecipitation results demonstrated that 4F2hc interacted with B3GNT3 in PANC-1 cells treated with or without RSL3 (Fig. S1L). We further confirmed that the protein expression of 4F2hc and B3GNT3 gradually increased in PANC-1 cells treated with RSL3 in a dose-dependent escalation manner (Fig. 1M). In contrast, the protein expression levels of xCT, DHODH, FSP1, and GPX4 showed the opposite trend (Fig. 1M). Finally, we confirmed that the protein levels of 4F2hc, B3GNT3, xCT, and GPX4 were highly expressed in PDAC cells compared to normal human pancreatic epithelial nestin-expressing (HPNE) cells (Fig. 1N). These results imply that the glycosyltransferase B3GNT3 could be a potential modulator in the response to ferroptosis, which might involve *N*-glycosylation of 4F2hc.

### Clinical significance of 4F2hc and B3GNT3 in PDAC patients

**Fig. 2:**
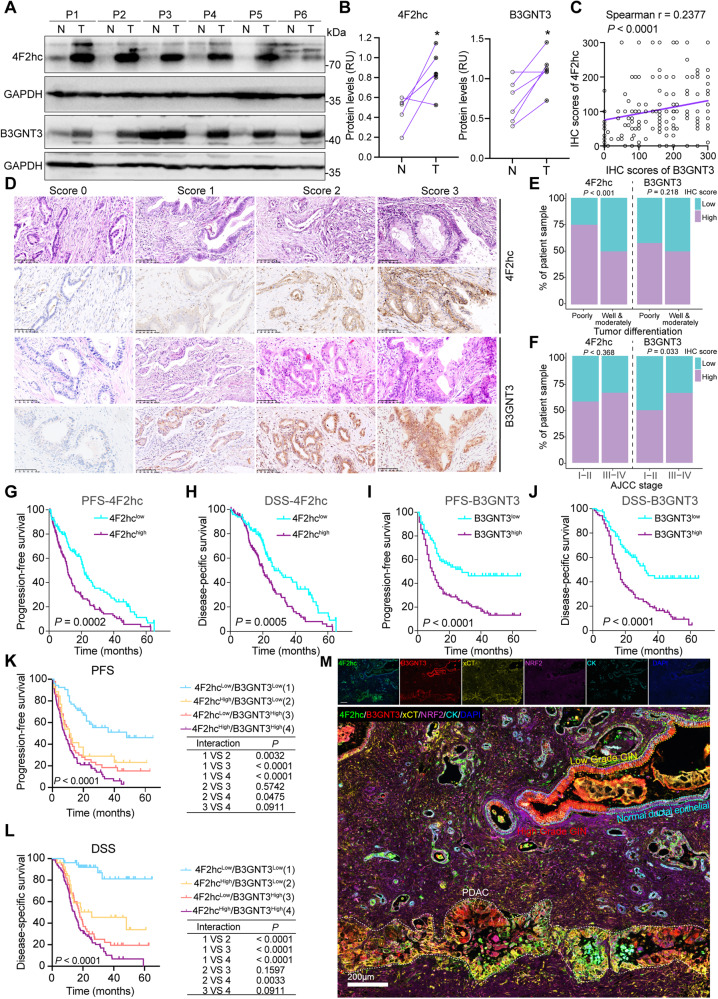
Association between 4F2hc and B3GNT3 expression and prognosis in a large cohort of PDAC patients. **A**. 4F2hc and B3GNT3 protein expression in paired tumor and adjacent normal tissues from 6 PDAC patients was determined by western blotting. **B** Paired comparison of 4F2hc and B3GNT3 protein expression. Statistical significance was determined by a two-tailed paired t-test. **C** Scatter plots showed the Pearson correlation of IHC scores of 4F2hc and B3GNT3. The purple line was a linear fit changed with the expression of 4F2hc and B3GNT3. **D** Representative HE (top) and IHC (bottom) stained sections of 4F2hc and B3GNT3 in PDAC (scale bar = 100 μm). Scores 0, 1, 2, and 3 represent negative, low, moderate, and high, respectively. **E**, **F** Percentage of patient samples for 4F2hc and B3GNT3 in tumor differentiation and AJCC stage. The correlation between the indicated molecule and the indicated clinicopathological characteristics was analyzed by the chi-square test. **G**, **I**, **K** Kaplan–Meier curves of progression‐free survival (PFS) stratified by 4F2hc (**G**) and B3GNT3 (**I**) and combined expression (**K**). **H**, **J**, **L** Kaplan–Meier curves of disease-specific survival (DSS) stratified by 4F2hc (**H**) and B3GNT3 (**J**) and combined expression (**L**). **M** Representative multiplex immunohistochemistry images of the spatial distribution and abundance for 4F2hc, B3GNT3, xCT, NRF2, CK, and DAPI in TMAs sections from case 242 of PDAC. Glandular intraepithelial neoplasia, (GIN). Scale bar: 200 µm. Statistical significance among the indicated groups was assessed by a one-tailed t-test, one-sided log-rank test, or Tarone-Ware test. *P < 0.05.

Inspired by these results, we sought to assess the clinical significance of 4F2hc and B3GNT3 in PDAC. We first confirmed that the protein levels of 4F2hc and B3GNT3 were highly expressed in six paired PDAC tissues compared to adjacent normal tissues (Fig. 2A, B), which is consistent with the gene expression analysis from TCGA (Fig. S2I, J). We further confirmed that 4F2hc and B3GNT3 were highly expressed (score of 2-3) in 59.92% (145/242) and 79.30% (203/256) of the 291 PDAC patient specimens, respectively (Fig. S2K, L). High expression of B3GNT3 positively correlated with 4F2hc in patients with PDAC (Fig. 2C). 4F2hc exhibited a membranous staining pattern, whereas B3GNT3 staining was visualized as tan granules scattered around the cytoplasm (Fig. 2D). In addition, the correlation analysis showed that high expression of 4F2hc was more frequent in PDAC with poor differentiation, and positive protein expression of B3GNT3 was more significantly associated with American Joint Committee on Cancer (AJCC) III-IV stage and tumor stage in T1-2 (Fig. 2E, F) (Table S3).

Notably, high protein expression of 4F2hc and B3GNT3 was significantly associated with poor progression-free survival (PFS) and disease-specific survival (DSS) (Fig. 2G–J), which is consistent with the univariate analysis (Table S4). Multivariate analyses indicated that the high protein expression of 4F2hc and B3GNT3 was an independent predictor of poor PFS and DSS (Table S5). Surprisingly, the prognostic value of 4F2hc and B3GNT3 could be markedly modified by a combination of their different levels of expression (Fig. 2K, L). Finally, multiplex immunohistochemistry characterized 4F2hc and B3GNT3 were detected in cytokeratin (CK)-positive epithelial cells, and both interacted spatially and were extensively mapped in similar areas. Remarkably, we observed a gradual increase in the expression of B3GNT3 with the progression of glandular intraepithelial neoplasia but slightly declined in PDAC (Fig. 2M, Fig. S2M, N). Taken together, these results imply that both 4F2hc and B3GNT3 contribute to PDAC development and prognosis.

### Abrogation of *N*-glycosylation of 4F2hc sensitizes PDAC cells to ferroptosis insult

**Fig. 3:**
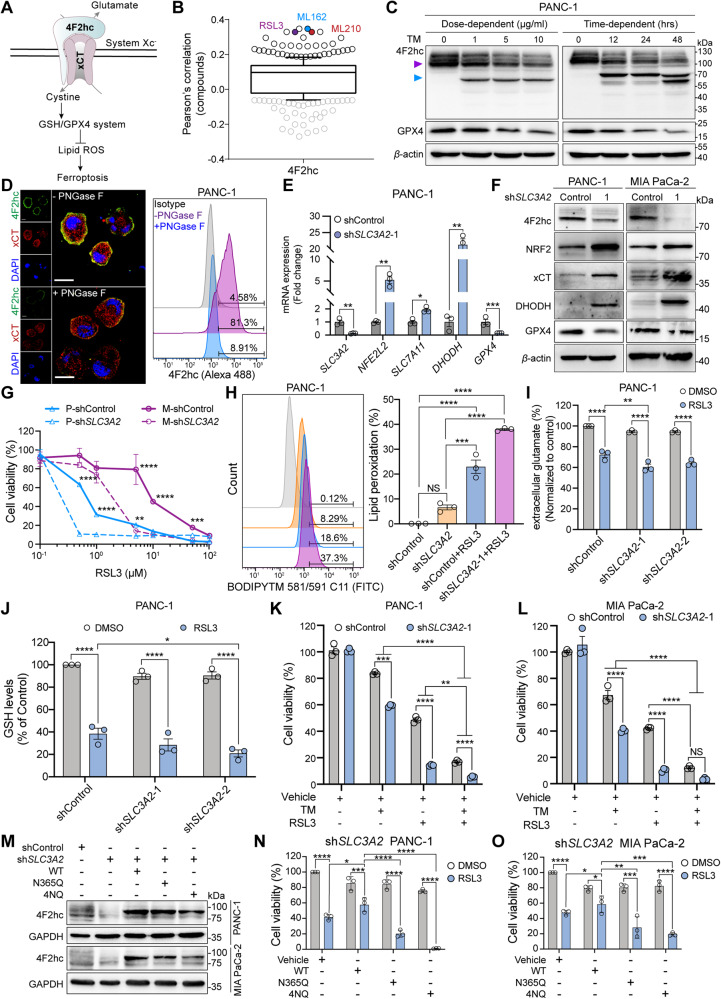
*N*-glycosylation of 4F2hc plays a critical role in the ferroptosis sensitivity of PDAC cells. **A** Schematic plot of the 4F2hc and xCT complex and its function. **B** High expression of 4F2hc shows a positive correlation with resistance to ferroptosis inducers (RSL3 (Pearson’s correlation coefficient (PCC) = 0.329), ML162 (PCC = 0.348), and ML210 (PCC = 0.33)) in cancer cells. Data mined from the Cancer Therapeutics Response Portal (CTRP) (http://www.broadinstitute.org/ctrp). Box and whisker plots indicate the 10th to 90th percentile range, and minimum and maximum values, each value of distributions are PCC on the y-axis. **C** PANC-1 cells were treated with TM at different doses for different amounts of time, and the expression changes in 4F2hc and GPX4 were determined by western blotting. The purple- and cyan- triangles represent high- and low- glycosylation, respectively. **D** The expression and localization of transmembrane protein 4F2hc in PANC-1 cells treated with or without PNGase F are determined by the confocal image on the left and flow cytometry on the right. **E** mRNA levels of the indicated gene in *SLC3A2* knockdown PANC-1 cells were quantified by qRT–PCR. **F** Measurement of 4F2hc, NRF2, xCT (CST#12691, 35 kDa), DHODH, and GPX4 protein expression levels in PANC-1 cells with or without 4F2hc knockdown. **G** Cell viability of sh*SLC3A2* PANC-1 and MIA PaCa-2 cells treated with a range of RSL3 doses for 12 h. **H** Lipid ROS levels in shControl and sh*SLC3A2* PANC-1 cells treated with or without 0.8 μM RSL3 for 8 h. BODIPY™ 581/591 C11 staining was used for FACS analysis in FITC channel. **I** Extracellular glutamate levels in shControl and sh*SLC3A2* PANC-1 cells treated with or without 0.8 μM RSL3 for 12 h. **J** Intracellular GSH levels in shControl and sh*SLC3A2* PANC-1 cells treated with or without 0.8 μM RSL3 for 12 h. **K**, **L** Cell viability in shControl and sh*SLC3A2* PANC-1 and MIA PaCa-2 cells treated with or without 10 μg/ml TM or 0.8 μM RSL3 for 12 h. **M** Western blotting analyzing the protein expression of 4F2hc in *SLC3A2* knockdown PANC-1 and MIA PaCa-2 cells transfected with wildtype (WT) 4F2hc, N365Q, and 4NQ plasmids, respectively. **N**, **O** Cell viability analysis of sh*SLC3A2* PANC-1 (**N**) and sh*SLC3A2* MIA PaCa-2 cells (**O**) transfected with WT, N365Q, and 4NQ plasmids, respectively, with or without 0.8 μM RSL3 treatment for 12 h. All data are shown as the mean ± SD. Statistical significance among the indicated groups was assessed by ANOVA analysis or unpaired Student’s t-test. At least three independent experiments except for B and D, 3 technical replicates for G. *P < 0.05, **P < 0.01, ***P < 0.001, ****P < 0.0001.

The interaction between 4F2hc and xCT hinders ferroptosis by increasing intracellular cystine influx and the subsequent build-up of the glutathione (GSH)/GPX4 system (Fig. 3A). By analyzing the Cancer Therapeutics Response Portal (CTRP) database, we found that the elevated gene expression of *SLC3A2* was positively correlated with resistance to GPX4 inhibitors such as RSL3, ML162, and ML210, especially in RLS3-treated pancreatic cancer cell lines (Fig. 3B, Fig. S3A, B). Notably, the expression of the high-glycosylation form of 4F2hc decreased gradually in a dose- and time-dependent manner of TM in PANC-1 cells and BxPC-3 cells, and the expression of GPX4 showed a similar decreasing trend (Fig. 3C, Fig. S3C, D). Furthermore, *N*-glycosylated 4F2hc disappeared from the cell membrane surface after treatment with PNGase F, and the expression of xCT was moderately reduced (Fig. 3D), suggesting that inhibition of *N*-glycosylation of 4F2hc could accelerate its glycoprotein degradation. Knockdown of *SLC3A2* (sh*SLC3A2*) activated the antioxidant system by upregulating the gene and protein expression of *NFE2L2*, *SLC7A11*, and *DHODH* but not GPX4 (Fig. 3E, F, Fig. S3E, F). sh*SLC3A2* markedly sensitized PANC-1 and MIA PaCa-2 cells to RSL3-induced ferroptosis (Fig. 3G), largely induced lipid peroxidation and decreased the amount of extracellular glutamate and intracellular GSH in RSL3-treated PANC-1 cells (Fig. 3H–J). Notably, TM treatment dramatically enhanced the sensitivity of PANC-1 and MIA PaCa-2 cells (regardless of *SLC3A2* knockdown or not) to RSL3-induced ferroptosis (Fig. 3K, L).

There are four *N*-glycosylation sites in human 4F2hc protein, namely Asn365, Asn381, Asn424, and Ans506, which are located in the extracellular domain of 4F2hc (Fig. S3G). To investigate the role of *N*-glycosylation of 4F2hc on ferroptosis resistance, a single Asn365 site mutant (asparagine (N) residue at 365 changed to glutamine (Q), N365Q) and four glycosylation sites mutants (4NQ) were constructed. Results confirmed that reconstitution of wildtype (WT) 4F2hc exhibited a much more pronounced protective effect against RSL3-induced ferroptosis in sh*SLC3A2* PANC-1 and MIA PaCa-2 cells. While N365Q and 4NQ mutants failed to restore the cell viability and showed greater sensitivity to RSL3-induced ferroptosis (Fig. 3M–O). Together, these results suggest that blocking the *N*-glycosylation of 4F2hc could sensitize PDAC cells to ferroptosis.

### Impaired B3GNT3 sensitizes PDAC cells to ferroptosis by regulating the stability of *N*-glycosylated 4F2hc

**Fig. 4:**
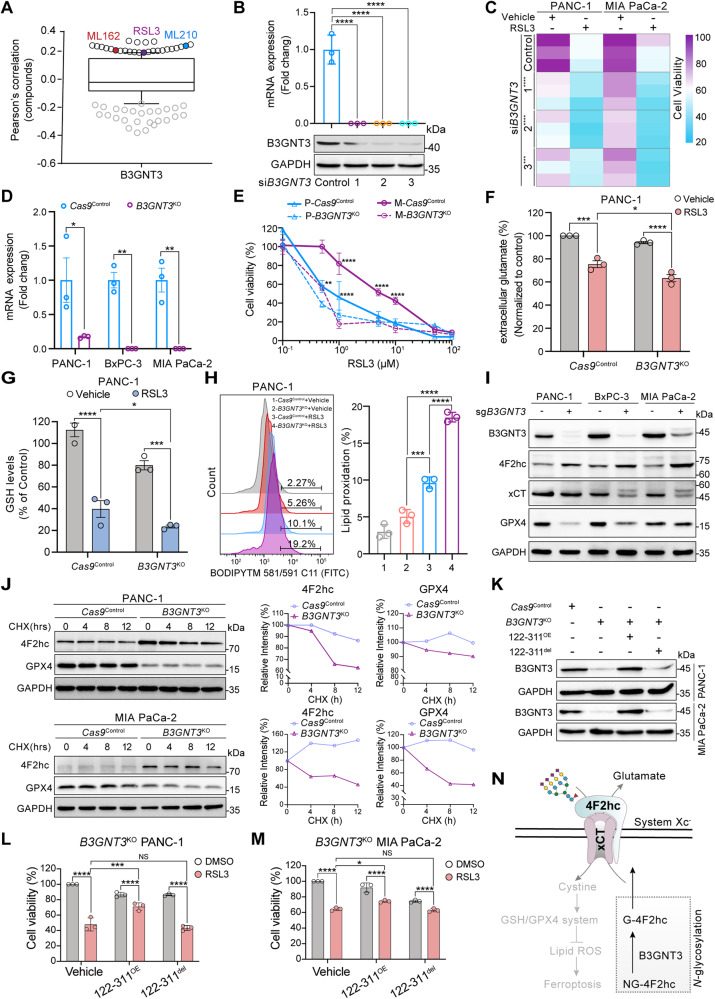
Deletion of the glycosyltransferase B3GNT3 enhances ferroptosis response in PDAC cells. **A** High expression of B3GNT3 shows a positive correlation with resistance to ferroptosis inducers (RSL3 (PCC = 0.19), ML162 (PCC = 0.206), and ML210 (PCC = 0.239)) in cancer cells. Data mined from the CTRP. **B** mRNA and protein levels of *B3GNT3* in si*B3GNT3* PANC-1 cells were quantified by qRT–PCR and western blotting. **C** Cell viability measurement of siControl and si*B3GNT3* PANC-1 and MIA PaCa-2 cells treated with 0.8 μM RSL3 for 12 h. **D** The mRNA expression levels of *B3GNT3* were detected in PANC-1, BxPC-3, and MIA PaCa-2 cells with indicated genotypes. **E** Cell viability measurement in *Cas9*^Control^ and *B3GNT3*^KO^ cell lines treated with a range of RSL3 doses for 12 h. **F**–**H** Measurement of extracellular glutamate (**F**), GSH (**G**), and lipid ROS (**H**) in *Cas9*^Control^ and *B3GNT3*^KO^ PANC-1 cells treated with 0.8 μM RSL3 for the indicated times. **I** Western blotting analyzing the protein expression of B3GNT3, 4F2hc, xCT, and GPX4 in PANC-1, BxPC-3, and MIA PaCa-2 cells with indicated genotypes. **J** CHX-chase analysis for PANC-1 and MIA PaCa-2 cells with the indicated genotypes. Cells were treated with 20 μM CHX at the indicated intervals, and then the expression of 4F2hc and GPX4 was measured by immunoblotting, and the intensity of 4F2hc and GPX4 was quantified and normalized to that at the 0-time point. **K** Western blotting analyzing the protein expression of B3GNT3 in *B3GNT3*^KO^ PANC-1 and MIA PaCa-2 cells transfected with 122-311^OE^ and 122-311^del^ plasmids, respectively. **L**, **M** Cell viability analysis of B3GNT3 knockout PANC-1 (**L**) and MIA PaCa-2 cells (**M**) transfected with 122-311^OE^ and 122-311^del^ plasmids, respectively, with or without 0.8 μM RSL3 treatment for 12 h. **N** The schematic diagram shows that B3GNT3 promotes the conversion of non-glycosylated (NG) 4F2hc into glycosylated (G) 4F2hc through *N*-glycosylation modification, then *N*-glycosylated 4F2hc translocate to the cell membrane to enhance the resistance of PDAC cells to ferroptosis. The western blotting experiment was representative of two biological replicates with similar results. All data are shown as the mean ± SD. Statistical significance among the indicated groups was assessed by ANOVA analysis or unpaired Student’s t-test. NS: no significance. *P < 0.05, **P < 0.01, ***P < 0.001, ****P < 0.0001.

Glycotransferase B3GNT3 was initially identified as an enzyme that catalyzes the glycosylation of proteins. However, its potential biological role in ferroptosis has not been reported. We first confirmed that high expression of *B3GNT3* was positively correlated with resistance to ferroptosis inducers including RSL3, ML162, and ML210, particularly in pancreatic cancer cell lines treated with RSL3 (Fig. 4A, Fig. S4A). Knockdown of *B3GNT3* markedly sensitized PANC-1 and MIA PaCa-2 cells to RSL3-induced ferroptosis (Fig. 4B, C). CRISPR-*Cas*9-mediated *B3GNT3* knockout (*B3GNT3*^KO^) markedly sensitized PANC-1 and MIA PaCa-2 cells to RSL3-induced ferroptosis (Fig. 4D, E), which could be largely rescued by ferroptosis inhibitors (such as Liproxstatin-1, UAMC, and vitamin E), but could not be reversed by apoptosis inhibitor (Z-VAD-FMK) and necroptosis inhibitor (Necrostatin-1) (Fig. S4B). *B3GNT3*^KO^ potently decreased the levels of extracellular glutamate and intracellular GSH (Fig. 4F, G) and triggered the accumulation of lipid peroxidation products in RSL3-challenged PANC-1 cells (Fig. 4H). Notably, we found that overexpression of *SLC3A2*, but not *B3GNT3*, largely rescued RSL3-induced ferroptosis in sh*SLC3A2* PDAC cells (Fig. S4C, D). We further confirmed that *B3GNT3*^KO^ substantially decreased the expression of xCT and GPX4, but compensatorily increased the expression of 4F2hc (Fig. 4I). To elucidate the underlying mechanism, we further investigated the effect of B3GNT3 on the protein stability of 4F2hc. The results showed that *B3GNT3*^KO^ accelerated the protein degradation of 4F2hc albeit with compensatory upregulation in PANC-1 and MIA PaCa-2 cells (Fig. 4J). Remarkably, the expression of GPX4 exhibited a faster turnover in *B3GNT3*^KO^ cells than in *Cas9*^Control^ cells (Fig. 4J), implying that there is an underlying mechanism for the quality control of GPX4 regulated by B3GNT3-mediated glycosylation.

Considering that *N*-glycosylation of 4F2hc catalyzed by B3GNT3 depends on its enzymatic activity, we constructed two plasmids for overexpression and deletion of the enzyme activity of B3GNT3, denoted as 122-311^OE^ and 122-311^del^, respectively (Fig. S4E). Results showed that 122–311^OE^ significantly delayed the protein attenuation of 4F2hc and GPX4 compared with the 122–311^del^ in *B3GNT3*^KO^ PANC-1 and MIA PaCa-2 cells (Fig. S4F, G), suggesting overexpression of enzymatically active B3GNT3 had a moderate protective effect on the protein expression of 4F2hc and GPX4 in PDAC cells. We further confirmed that reconstitution of enzymatically active 122-311^OE^ partially restored the cell viability in *B3GNT3*^KO^ PANC-1 and MIA PaCa-2 cells treated with RSL3, while reconstitution with a glycosyltransferase-null 122–311^del^ constructs could not (Fig. 4K–M). Together, our data suggest that B3GNT3 promotes the formation of non-glycosylated (NG) 4F2hc into glycosylated (G) 4F2hc through *N*-glycosylation modification which stabilizes *N*-glycosylated 4F2hc, thereby enhancing 4F2hc-mediated ferroptosis resistance (Fig. 4N).

### *N*-glycosylation stabilizes 4F2hc in PDAC cells

**Fig. 5:**
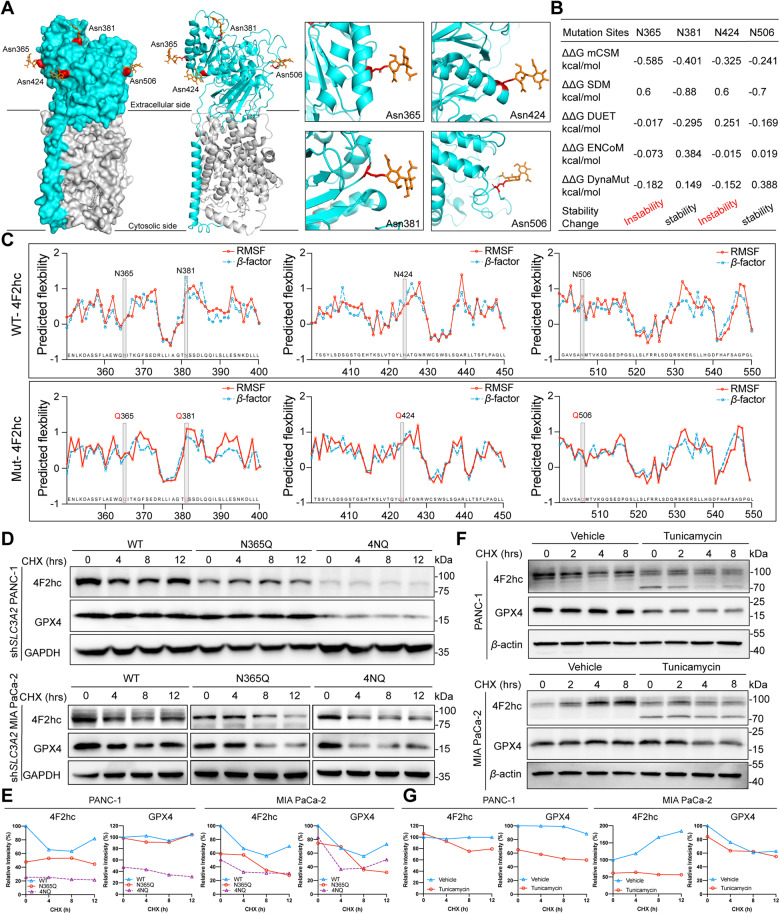
*N*-glycosylation of 4F2hc is critical for its protein stability. **A** Structure of human Erastin-bound 4F2hc-xCT complex (PDB: 7EPZ), shown as surface (left) and cartoon (middle). Detailed view of four glycosylation sites, shown as red sticks (right). Cyan denotes 4F2hc, gray denotes xCT, and orange denotes carbohydrate. **B** Prediction of protein stability changes upon *N*-glycosylation sites mutation in 4F2hc using DynaMut (https://biosig.lab.uq.edu.au/dynamut/). **C** Prediction of the impact of glycosylation site mutations of 4F2hc on its protein flexibility and stability by PredyFlexy (one available online software for predicting root mean square fluctuations (RMSF) and *β*-factor based on MD simulations) (https://www.dsimb.inserm.fr/dsimb_tools/predyflexy/). Red and blue lines represent the RMSF and *β*-factor prediction respectively deduced from flexibility class prediction based on MD simulation. The higher value of RMSF or *β*-factor after mutation of a glycosylation site indicates that a residue is more rigid and vice versa. **D** Western blot analyzing the protein expression of 4F2hc and GPX4 in sh*SLC3A2* PANC-1 and MIA PaCa-2 cells reconstituted with N365Q and 4NQ mutant after treated with CHX (20 μM) at indicated intervals. **E** The intensity of 4F2hc and GPX4 was quantified using ImageJ software and values were normalized to that at the 0-time point for comparative analysis. **F** PANC-1 and MIA PaCa-2 cells treated with 20 μM CHX at the indicated intervals in the presence of 10 μg/ml tunicamycin or not. **G** Quantification of indicated proteins using ImageJ software and values were normalized to that at the 0-time point for comparative analysis.

The crystal structure of the Erastin-bound 4F2hc-xCT complex (PDB: 7EPZ) was characterized very recently (Fig. 5A). Previous studies have shown that removing *N*-glycan from a glycoprotein can lead to structural instability. However, whether *N*-glycosylation affects the stability of 4F2hc has not been further inspected. To do this, the *N*-glycosylation sites of 4F2hc were mutated and their changes in protein stability and flexibility were predicted by DynaMut and PredyFlexy based on MD simulations. Results showed that mutations of Asn365 and Asn424 led to instability of 4F2hc protein, manifested by reduction of the Gibbs free energy (ΔΔG) (Fig. 5B). The flexibilities (root-mean-square fluctuations (RMSFs)) were moderately elevated when glycosylation sites N365, N381, and N424 were mutated, indicating that the mutation of *N*-glycosylation sites of 4F2hc increased its structural fluctuation which may lead to instability of 4F2hc protein (Fig. 5C). Differential *N*-glycosylation site Asn365 in 4F2hc was conserved across multiple species (Fig. S5A). Since the *N*-glycosylation of 4F2hc mediated by B3GNT3 is essential for its protein stability and the establishment of the xCT-GPX4-involved antioxidant system, we confirmed that re-expression of *N*-glycosylation mutant form N365Q and 4NQ significantly decreased the protein expression of xCT, GPX4, and B3GNT3 in sh*SLC3A2* PANC-1 cells (Fig. S5B). This is probably due to the deglycosylation of 4F2hc accelerated its protein instability and attenuation, reduced its demand for glycosyltransferase B3GNT3, downregulated the protein expression of its chaperone xCT, and ultimately decreased the expression of downstream GPX4. We further confirmed that reconstitution of N365Q and 4NQ mutants accelerated the protein degradation rate of 4F2hc and GPX4 in sh*SLC3A2* PANC-1 and MIA PaCa-2 cells treated with protein synthesis inhibitor cycloheximide (CHX) for different times (Fig. 5D, E), suggesting that deglycosylated 4F2hc proteins are unstable and presumably more susceptible to degradation. Moreover, CHX-chase assay revealed that the expression of less-glycosylated 4F2hc was faster decreased than high-glycosylated 4F2hc in PANC-1 and MIA PaCa-2 cells upon *N*-linked glycosylation inhibitor tunicamycin (TM) treatment (Fig. 5F, G). Similarly, GPX4 exhibited a faster degradation rate in TM-treated PANC-1 and MIA PaCa-2 cells. In conclusion, those data suggest that *N*-glycosylation of 4F2hc is responsible for 4F2hc protein stability.

### *N-*glycosylation of 4F2hc is required for its membrane localization and the interaction with xCT

**Fig. 6:**
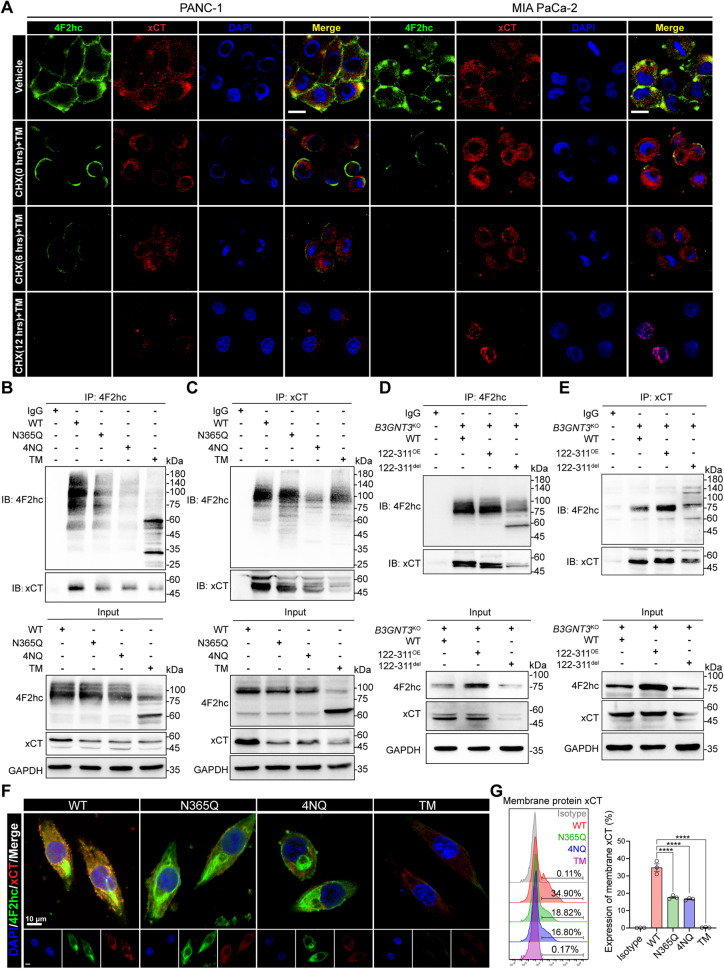
*N*-glycosylation of 4F2hc is required for its membrane localization and the combination with xCT. **A** Laser scanning confocal microscope (LSCM) images of PANC-1 and MIA PaCa-2 cells after treatment with 10 µg/ml TM for 12 h and then incubation with CHX for 0, 6, and 12 h. 4F2hc, xCT, and DAPI were stained with Alexa Fluor^®^ 488, Alexa Fluor^®^ 594, and 4′,6-diamidino-2′-phenylindole dihydrochloride, respectively. Scale bar, 20 μm. **B**, **C** Co-IP analysis of the effect of de-glycosylation mutants N365Q and 4NQ on the interaction between 4F2hc and xCT (Abcam#175186, 55 kDa) in sh*SLC3A2* PANC-1 cells. **D**, **E** Co-IP analysis of the interaction between 4F2hc and xCT (Abcam#175186, 55 kDa) in *B3GNT3*^KO^ PANC-1 cells reconstituted with 122-311^OE^ and 122-311^del^ plasmids. **F** The confocal image determines the co-localization of transmembrane protein 4F2hc and xCT in sh*SLC3A2* MIA PaCa-2 cells transfected with WT, N365Q, or 4NQ or in MIA PaCa-2 cells treated with TM. Scale bar, 10 μm. **G** Flow cytometry examines the membrane expression of xCT in sh*SLC3A2* MIA PaCa-2 cells transfected with WT, N365Q, or 4NQ or in MIA PaCa-2 cells treated with TM.

Aberrant *N*-glycosylation of a protein alters the interaction with its partner, leading to inappropriate membrane localization of its chaperone [14]. Given that xCT fixed at the cell surface depends on a short helix (H1′) formed by the residues in 4F2hc [8], we wondered whether de-glycosylation of 4F2hc affects its cell membrane location and the interaction with xCT. First, the co-expression and membrane colocalization of 4F2hc and xCT were observed in wild-type parental PANC-1 and MIA PaCa-2 cells but noticeably decreased with prolonged CHX treatment time in TM-challenged cells (Fig. 6A). In addition, the fluorescence intensity of 4F2hc was enhanced in RSL3-challenged PANC-1 cells but its expression and membrane colocalization with xCT were largely quenched in TM-treated PANC-1 cells with or without RSL3 co-treatment (Fig. S5C). Moreover, the upregulation of 4F2hc induced by RSL3 was mitigated by the treatment of TM in PANC-1 cells, and the expression of NRF2, xCT, and B3GNT3 was decreased as well (Fig. S5D). The Co-IP results demonstrated that the knockdown of *SLC3A2* indeed blocked the interaction between 4F2hc and xCT, this physiological interaction was also disrupted in the TM-treated PANC-1 cells (Fig. S5E, F). We further confirmed that de-glycosylation of 4F2hc by re-expressing of N365Q or 4NQ or treating with TM obviously decreased the interaction between 4F2hc and xCT in sh*SLC3A2* PANC-1 cells (Fig. 6B, C**)**. In addition, we confirmed that reconstitution with enzymatically active 122-311^OE^ plasmids retained the interaction between 4F2hc and xCT, while reconstitution with a glycosyltransferase-dead 122-311^del^ mutant decreased this interplay in *B3GNT3*^KO^ PANC-1 cells (Fig. 6D, E**)**. Notably, we found that N365Q or 4NQ or TM treatment obviously alleviated the colocalization of 4F2hc and xCT in sh*SLC3A2* MIA PaCa-2 cells, and the membrane protein expression level of xCT decreased significantly (Fig. 6F, G). We further confirmed that reconstitution of glycosylation-resistant N365Q or 4NQ mutant did not rescue the membrane expression of 4F2hc and the colocalization with xCT in 4F2hc-deficient PANC-1 cells (Fig. S5G, H). In summary, blocking the *N*-glycosylation of 4F2hc attenuates its membrane expression and the interaction with xCT, thereby conferring sensitivity to ferroptosis in PDAC cells.

### TM exposure increased the sensitivity of PDAC cells to ferroptosis partially by suppressing *N*-glycosylation

**Fig. 7:**
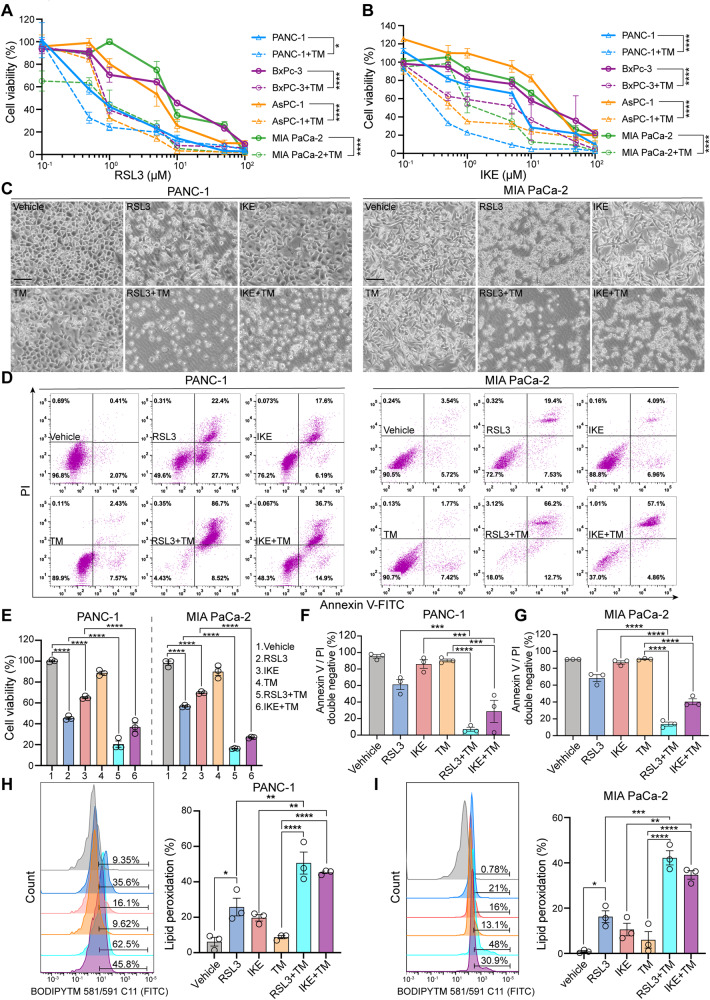
Treatment with *N*-glycosylation inhibitor TM largely sensitized PDAC cells to ferroptosis. **A**, **B** Cell viability analysis of PDAC cells treated with RSL3 and IKE when combined with or without TM. **C–I** PANC-1, and MIA PaCa-2 cells were treated with 0.8 μM RSL3, 20 μM IKE, 10 μg/ml TM, RSL3 + TM, or IKE + TM for 12 h, respectively. Representative cell death images are shown in (**C**). Scale bar, 50 μm. **D**, **F**, **G** Apoptotic assay by FITC Annexin V/PI staining, primary FACS plots (**D**), and quantification diagram (**F**, **G**) are displayed. **E** Cell viability analysis. **H**, **I** Lipid ROS were stained with BODIPY™ 581/591 C11, and the levels were assayed by FACS in the FITC channel. All data are shown as the mean ± SD. Statistical significance among indicated groups was assessed by ANOVA analysis or unpaired Student’s t-test. *P < 0.05, **P < 0.01, ***P < 0.001, ****P < 0.0001.

Given the above findings, we aim to investigate the potential therapeutic values of TM in PDAC cells and evaluate whether the sensitivity of PDAC cells to ferroptosis inducer could be largely enhanced by TM. Results showed that four human PDAC cell lines treated with TM were more sensitive to either RSL3 or imidazole ketone erastin (IKE)-induced ferroptosis than those without TM treatment (Fig. 7A, B). Similarly, the cell viability of PANC-1 and MIA PaCa-2 cells was largely decreased under the combination treatment with TM plus RSL3 or IKE (Fig. 7C, E). In addition, we observed relatively fewer annexin V/PI double-negative cells in indicated cells challenged with TM plus RSL3 or IKE than in RSL3- or IKE-treated cells individually (Fig. 7D, F, G). Furthermore, the levels of lipid peroxidation products were remarkably increased in PANC-1 and MIA PaCa-2 cells treated with TM plus RSL3 or IKE compared to those in RSL3- or IKE-treated counterparts (Fig. 7H, I). Gemcitabine (GEM) is a first-line chemotherapy regimen for pancreatic cancer but its clinical effect is limited owing to chemoresistance [30, 31]. To investigate whether the sensitivity of PDAC to GEM can be largely improved by combining with ferroptosis inducer RSL3 or glycosylation inhibitor TM, we confirmed that different concentrations of GEM plus RSL3 or TM significantly reduced the viability of PANC-1 cells compared to that in the monotherapy group, and GEM plus RSL3 plus TM was lethal to PANC-1 cells at a lower concentration than either drug alone **(**Fig. S6A, B**)**. Additionally, sh*SLC3A2* or treated with TM greatly reduced the cell viability of GEM-challenged PANC-1 cells (Fig. S6C, D). Together, our results suggest that targeting the *N*-glycosylation pathway holds great potential for the combination therapy of PDAC.

### Knockout of *B3GNT3* limited the proliferative capacity of PDAC cells in vitro and in vivo

**Fig. 8:**
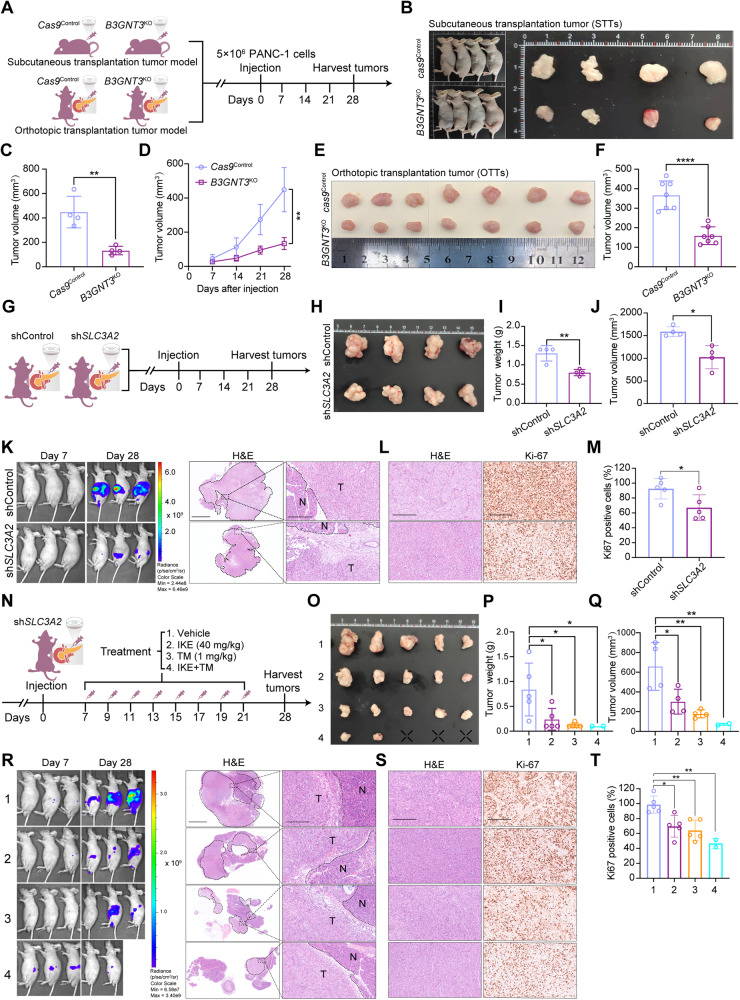
Knockout of *B3GNT3* or knockdown *SLC3A2* retarded PDAC cell growth and potentiated IKE or TM-mediated PDAC suppression in vitro and in vivo. **A** Schematic of the experimental design for nude mice bearing PANC-1 xenografts with the indicated genotypes. **B** Representative subcutaneous transplant tumors (STTs) were removed and photographed on day 28 (n = 4 mice/group). **C** Relative tumor volume of STTs. **D** The tumor growth curves of STTs were drawn and assessed weekly. **E** Gross appearances of representative orthotopic transplantation tumors (OTTs) were removed and photographed on day 28 (n = 7 mice/group). **F** Relative tumor volume of OTTs. For detailed methods of STTs and OTTs experiments, see the methods section. **G** Schematic of the experimental design for nude mice bearing PANC-1 cells stably transfected with shControl and sh*SLC3A2*. **H** Representative orthotopic transplantation tumors (OTTs) were removed and photographed on day 28 (n = 4 mice/group). **I** Tumor weight. **J** Tumor volume. **K** Left, representative IVIS bioluminescence images on day 7 and day 28; right, representative hematoxylin and eosin (HE)-stained tumor section. Scale bar, 2.5 mm for zoom out and 250 μm for zoom in. N, normal tissue. T, tumor. **L** Left, representative HE sections with corresponding immunohistochemical staining for Ki-67, Scale bar, 250 μm. **M** Percentage of Ki-67 positive stained cells per field at least 5 random fields were selected. **N** Treatment scheme for OTT nude mice bearing sh*SLC3A2* PANC-1 cells xenograft. BALB/c nude mice were randomly divided into four groups and injected intraperitoneally with the vehicle, 40 mg/kg IKE, 1 mg/kg TM, or the combination of IKE and TM once every other day. **O** Gross appearances of representative OTT were removed and photographed on day 28 (n = 5 mice/group). **P** Tumor weight. **Q** Tumor volume. **R** Left, representative IVIS bioluminescence images on day 7 and day 28; right, representative HE-stained tumor section. Scale bar, 2.5 mm for zoom out and 250 μm for zoom in. N, normal tissue. T, tumor. **S** Left, representative HE sections with corresponding immunohistochemical staining for Ki-67, Scale bar, 250 μm. **T** Percentage of Ki-67 positive stained cells per field at least 5 random fields were selected. All data are shown as the mean ± SD. Statistical significance among the indicated groups was assessed by ANOVA analysis or unpaired Student’s t-test. *P < 0.05, **P < 0.01, ****P < 0.0001.

To investigate whether *B3GNT3* affects the cell biological behavior of PDAC cells, scratch assays were performed and results showed that *B3GNT3*^KO^ markedly attenuated the wound closure in PANC-1 and MIA PaCa-2 cells compared to that in wild-type cells (Fig. S7A). Transwell assay showed a noticeable reduction in the number of migration and invasion of *B3GNT3*^KO^ PANC-1 and MIA PaCa-2 cells (Fig. S7B). Similarly, *B3GNT3*^KO^ suppressed the colony-forming abilities of PANC-1 and MIA PaCa-2 cells (Fig. S7C). To further investigate whether B3GNT3 promotes the progression of PDAC depending on its glycosyltransferase activity, *B3GNT3* knockout cells reconstituted with a glycosyltransferase-dead 122-311^del^ mutant were used in this concern. Results showed that there has no significant difference in the clone formation, wound closure, migration, and invasion in *B3GNT3*^KO^ PANC-1 and MIA PaCa-2 cells reconstituted with or without glycosyltransferase-null 122-311^del^ mutant, suggesting that glycosyltransferase activity of B3GNT3 plays an important role for PDAC cells proliferation (Fig. S8A–C). We next investigated the potential anticancer activity of *B3GNT3*^KO^ in subcutaneous transplant tumors (STTs) and orthotopic transplantation tumors (OTTs) implanted with PANC-1 cells (Fig. 8A). The results showed that *B3GNT3*^KO^ markedly slowed the growth of STTs and OTTs compared to normal counterpart as indicated by the tumor volume and tumor growth curves (Fig. 8B–F). These results suggested that *B3GNT3* may be a crucial gene mainly involved in PDAC progression.

### *SLC3A2* knockdown retards PDAC cell growth and potentiates IKE or TM-mediated PDAC suppression

4F2hc, as an important chaperone in amino acid transporter, few studies have reported its role in PDAC. We first confirmed that *SLC3A2* knockdown significantly suppressed wound closure in PANC-1 and MIA PaCa-2 cells (Fig. S7D). Likewise, the transwell assay showed that the migration and invasion of sh*SLC3A2* PANC-1 and MIA PaCa-2 cells were remarkably inhibited (Fig. S7E). Relative colony formation ability was hampered in sh*SLC3A2* PANC-1 and MIA PaCa-2 cells (Fig. S7F). Additionally, the OTTs model confirmed that knockdown of *SLC3A2* slowed the tumor growth (Fig. 8G, H), reduced the tumor weight and tumor volume **(**Fig. 8I, J), and had no effect on mouse weight (Fig. S8D). This is also supported by the bioluminescent signals, HE staining (Fig. 8K), and the percentage of Ki-67 positive cells (Fig. 8L–M). Notably, this tumor suppression effect could be further dramatically potentiated after treatment with IKE or TM **(**Fig. 8N–R**)**. The combination of IKE and TM largely inhibited the tumor growth in sh*SLC3A2* OTTs but caused undesirable toxicity manifested as significant mice weight loss **(**Fig. 8O–R, S8E). Additionally, Ki-67 staining showed the same trend as that mentioned above (Fig. 8S, T). Taken together, these results indicated that knockdown of *SLC3A2* contributes to PDAC tumor suppression and this antitumor activity can be largely enhanced by combination with IKE or TM.

## Discussion

Aberrant glycosylation involves multifaceted pathological and pathophysiological changes in PDAC, including but not limited to conferring tumor cells the ability to resist cell death. Ferroptosis and glycosylation are both physiological metabolic processes and provide potential opportunities to target such vulnerabilities for PDAC treatment [32]. However, their crosstalk remains unclear. In this study, we provided functional experimental and clinical evidence supporting an interesting and previously unexplored mechanism of *N*-glycosylation of 4F2hc in PDAC ferroptosis. In the context of PDAC, (i) aberrant *N*-glycosylation of 4F2hc is catalyzed by glycosyltransferases B3GNT3, which stabilizes the *N*-glycosylated 4F2hc localization on the cell surface and recruits xCT trafficking to the plasma membrane. Then, 4F2hc combined with xCT to support the proper assembly of system Xc^–^, which promotes the uptake of cystine and subsequent activation of the GSH-GPX4 axis, eventually enhancing PDAC to ferroptosis resistance. (ii) In contrast, deglycosylation of 4F2hc through PNGase F, protein *N*-glycosylation inhibitor TM, or *B3GNT3*^KO^ accelerates 4F2hc degradation and impairs its membrane localization, limiting the membrane trafficking of xCT and potentially disrupts system Xc^–^ assembly, ultimately promoting PDAC sensitivity to ferroptosis (Fig. S9).

Emerging evidence has suggested that metabolic reprogramming can confer ferroptosis resistance in malignancies [26, 33, 34]. The GPX4-GSH system functions primarily in antioxidant defense through system X_c_^–^ mediated uptake of cystine. However, most studies have only focused on xCT as a key transporter for cysteine, and have ignored the important prerequisite that the localization of xCT on the cell membrane requires the recruitment of 4F2hc [4, 35]. A recent study pointed out that 4F2hc plays an equally essential role as same as xCT for preventing YTHDC2-induced ferroptosis [36]. Notably, high expression of 4F2hc was positively correlated with ferroptosis resistance in the CTRP analysis. 4F2hc has four glycosylation sites [37, 38], we identified Asn365 as the most significant differential *N*-glycosylation site in RSL3-treated PANC-1 cells. This finding suggests that the ferroptosis stress-induced upregulation of the Asn365 glycosylation site of 4F2hc might be responsible for 4F2hc stabilization. However, few studies have reported the role of *N*-glycosylation of 4F2hc in PDAC ferroptosis. Thus, 4F2hc was the focus of our study. Protein glycosylation may alter the protein-protein interactions or affect the correct assembly of protein complexes [39]. Highlighted by the migration of molecular weight and the attenuation of protein stability of 4F2hc in PDAC cell lines treated with PNGase F and TM, we found the abrogation of *N*-glycosylation of 4F2hc or knockdown of *SLC3A2* inhibited the activity of system X_c_^–^ and largely sensitized PDAC cells to ferroptosis insults. Notably, we observed that the membrane colocalization of 4F2hc and xCT disappeared in TM-treated PANC-1 cells. Treatment with TM or knockdown of *SLC3A2* both significantly inhibited the interaction between 4F2hc and xCT. This may symbolize the dysfunction of system X_c_^–^ and is consistent with the notion that 4F2hc acts as a chaperone protein to support the function of xCT [7, 36]. Thus, we propose that *N*-glycosylated 4F2hc is critical for the full functionality of system X_c_^–^ and that targeting this pathway may increase the susceptibility of PDAC to ferroptosis.

*N*-glycosylation contributes to tumor heterogeneity and allows tumor cells to resist cell death induced by chemotherapy [40]. In our study, we found that 77.64% of *N*-linked glycoproteins were upregulated as compared to their *O*-linked counterparts in RSL3-induced PANC-1 cells. This indicates that *N*-glycosylation modifications are functionally significant for ferroptosis and require further in-depth investigation. Interestingly, GPX4, a vital intracellular detoxifying enzyme, its expression was significantly decreased in the TM-mediated CHX-chase assay, suggesting that the membrane glycoprotein GPX4 undergoes glycosylation, which is consistent with a previous study [41]. Further investigation of the glycosylation role of GPX4 in ferroptosis may be meaningful and warranted. The modification, processing, and biosynthesis of glycoproteins depends on the participation of glycosyltransferases [42]. B3GNT3 was first identified as a type II transmembrane protein involved in the glycosylation process [43]. High expression of B3GNT3 facilitates tumorigenesis and progression and is commonly associated with unfavorable survival in PDAC [44] and lung adenocarcinoma [45], but not in neuroblastoma [46]. Similarly, our study revealed that *B3GNT3*^KO^ suppressed PDAC cell proliferation, migration, invasion, and tumor suppression. Notably, we are the first to show that B3GNT3 plays a role in ferroptosis resistance partially by glycosylating 4F2hc.

TM was initially identified as a canonical inhibitor of *N*-linked glycosylation that disrupts UDP-*N*-acetylglucosamine (GlcNAc) transfer to dolichol phosphate in the ER thus affecting the modification, location, and function of glycoproteins and triggering ER stress [47, 48]. We found that TM treatment markedly increased the sensitivity of PDAC cells to RSL3 and IKE in vitro and in vivo. Although the role of ER stress induced by TM cannot be neglected, it is worth noting that most of the roles played by TM are initially dependent on the targeting of GlcNAc which catalyzes the committed step of *N*-glycosylation in the ER [48]. In other words, TM sensitized PDAC to ferroptosis at least in part by blocking *N*-glycosylation. Importantly, our findings established a potential link between ferroptosis and glycosylation by canonical *N*-glycosylation inhibitor TM. Furthermore, as a promising anticancer compound, TM could markedly enhance the sensitivity of cancer cells to radiation therapy and chemotherapy [49–51]. In our study, we found that either IKE or TM alone, or a combination of both, could significantly inhibit tumor growth in sh*SLC3A2* OTTs albeit with cytotoxicity caused by off-target inhibition of the *N*-glycosylation process mediated by TM [52]. Accordingly, further exploration of more specific *N*-glycosylation inhibitors or structural modifications to reduce the potential toxicity of TM may have extensive implications to attenuate ferroptosis resistance and increase the effectiveness of anti-PDAC therapeutics.

In summary, the data from our study demonstrate that the modulation of *N*-glycosylation of 4F2hc through the use of the *N*-glycosylation inhibitor TM or knockout of glycosyltransferase B3GNT3 can effectively increase the sensitivity of PDAC cells to ferroptosis. This finding suggests that targeting the B3GNT3-4F2hc-*N*-glycosylation pathway, specifically in PDAC, holds promise as a potential strategy to enhance the susceptibility of PDAC cells to ferroptosis.

## Supplementary information

Supplementary Figure 1

Supplementary Figure 2

Supplementary Figure 3

Supplementary Figure 4

Supplementary Figure 5

Supplementary Figure 6

Supplementary Figure 7

Supplementary Figure 8

Supplementary Figure 9

Supplementary Table 1

Supplementary Table 2

Supplementary Table 3

Supplementary Table 4

Supplementary Table 5

Supplementary Figure and Table Legends

Supplementary Materials and Methods

Original File of Western Bolts

## Data Availability

The RNA sequencing raw data have been deposited in the Gene Expression Omnibus (GEO) database with the accession number: GSE207741. Uncropped original western blots and relevant data are provided in the Supplementary files. The data generated in this study are available upon reasonable request from the corresponding author.
