# Supplementary figures and images for: Targeting N-glycosylation of 4F2hc mediated by glycosyltransferase B3GNT3 sensitizes ferroptosis of pancreatic ductal adenocarcinoma
Source: Cell Death Differ. 2023 Jul 21;30(8):1988–2004. doi: 10.1038/s41418-023-01188-z (PMC10406883; doi:10.1038/s41418-023-01188-z)

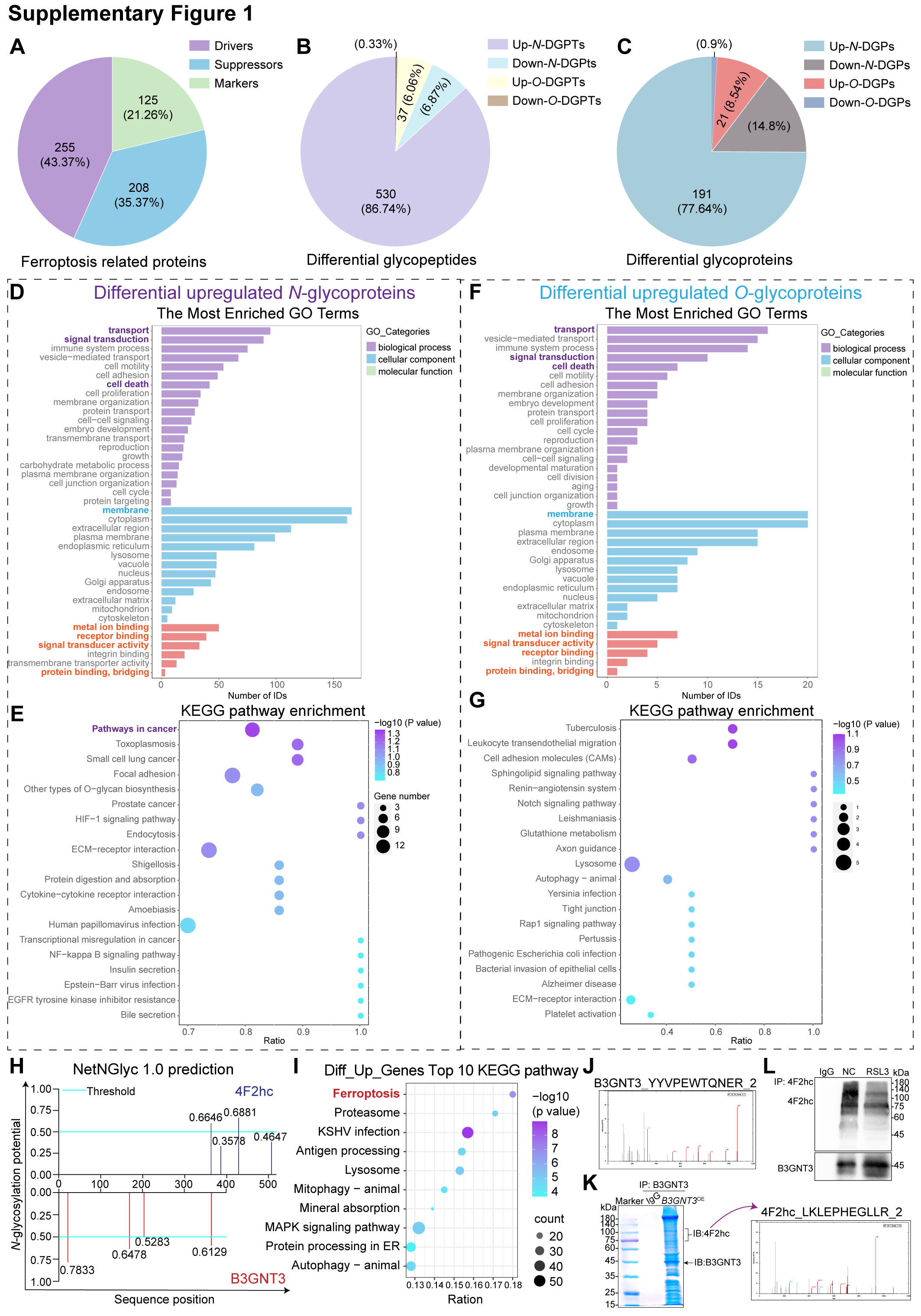

Supplement: Supplementary file 1 — Supplementary Figure 1 [file 41418_2023_1188_MOESM1_ESM.tif]

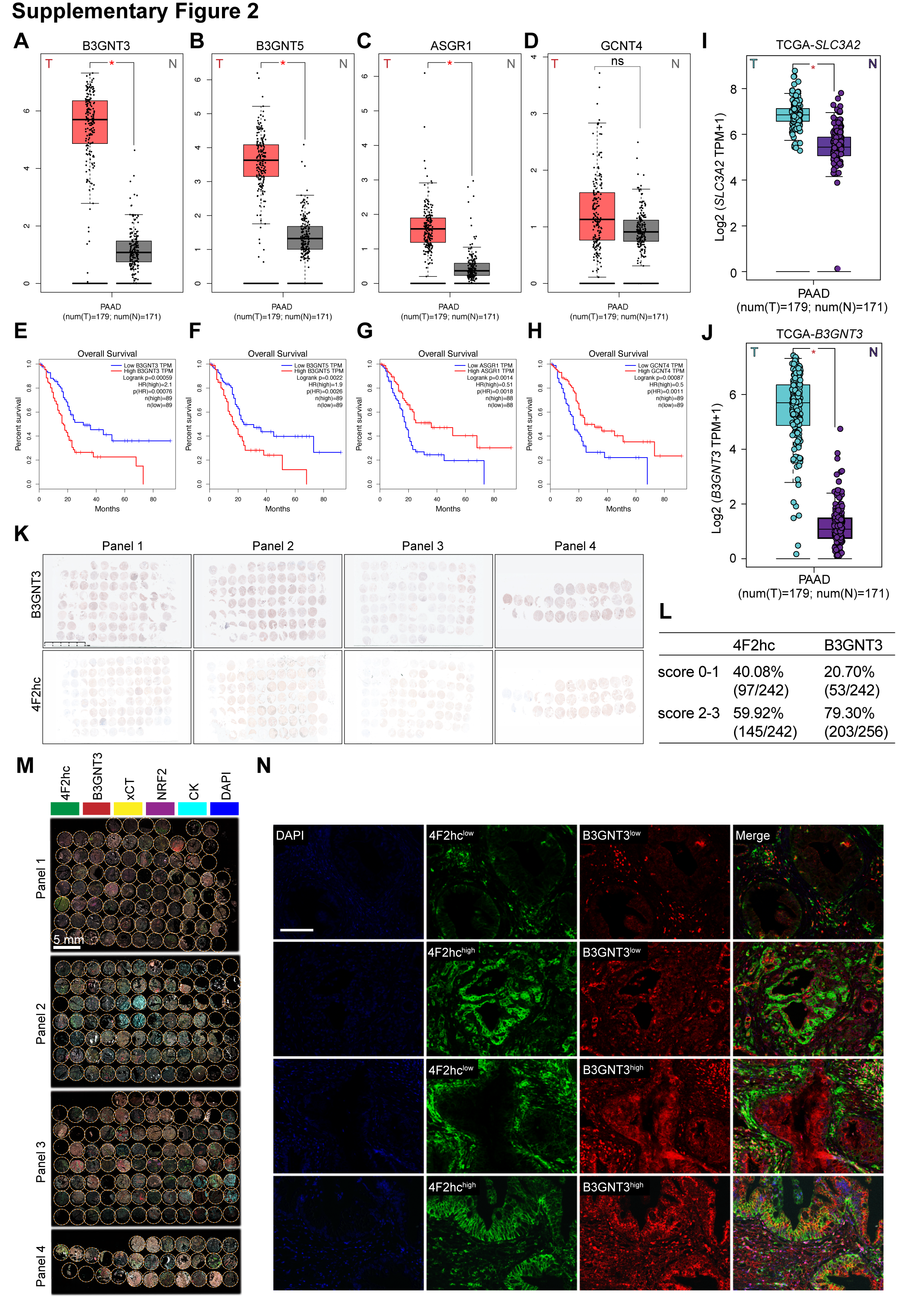

Supplement: Supplementary file 2 — Supplementary Figure 2 [file 41418_2023_1188_MOESM2_ESM.tif]

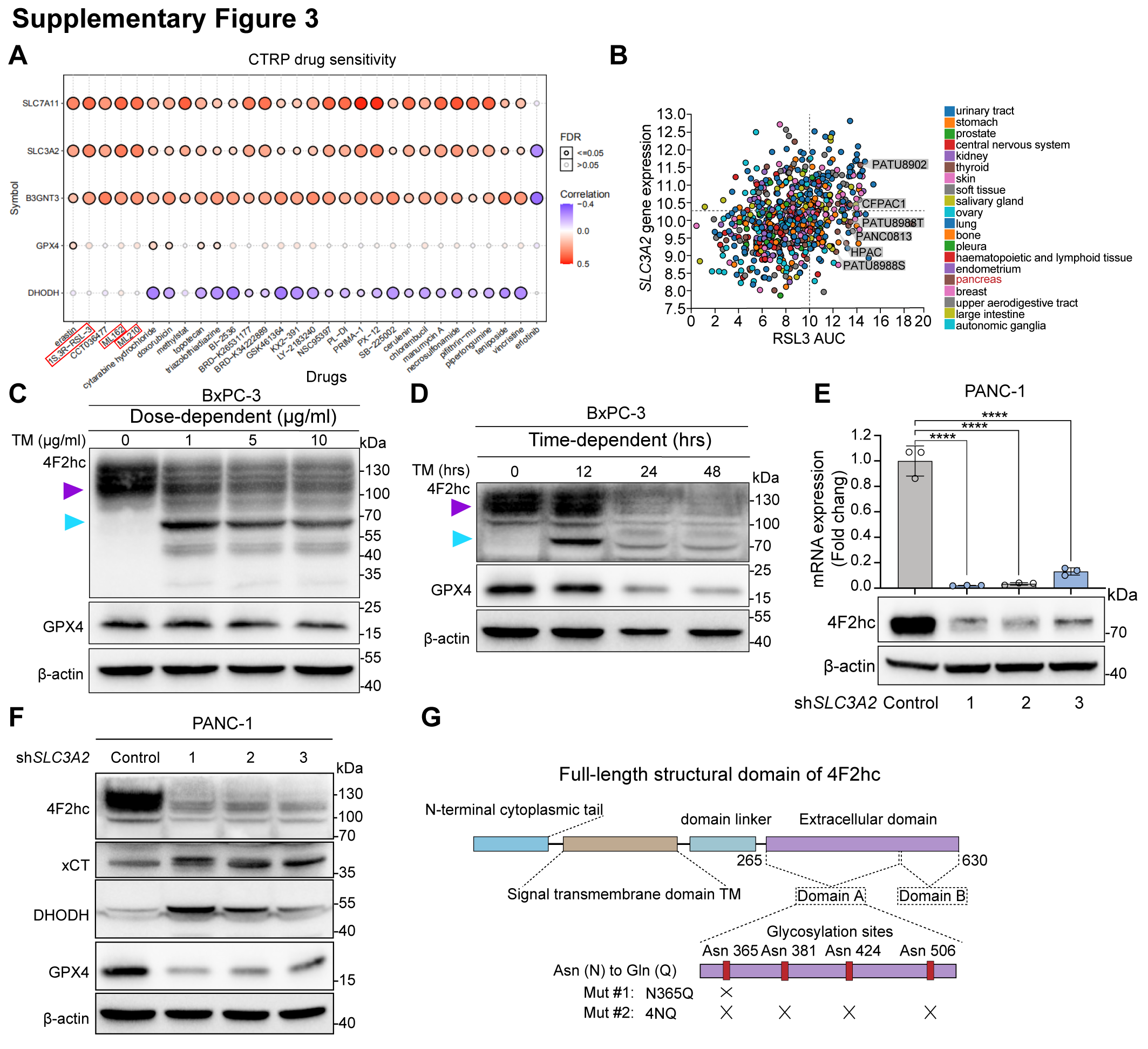

Supplement: Supplementary file 3 — Supplementary Figure 3 [file 41418_2023_1188_MOESM3_ESM.tif]

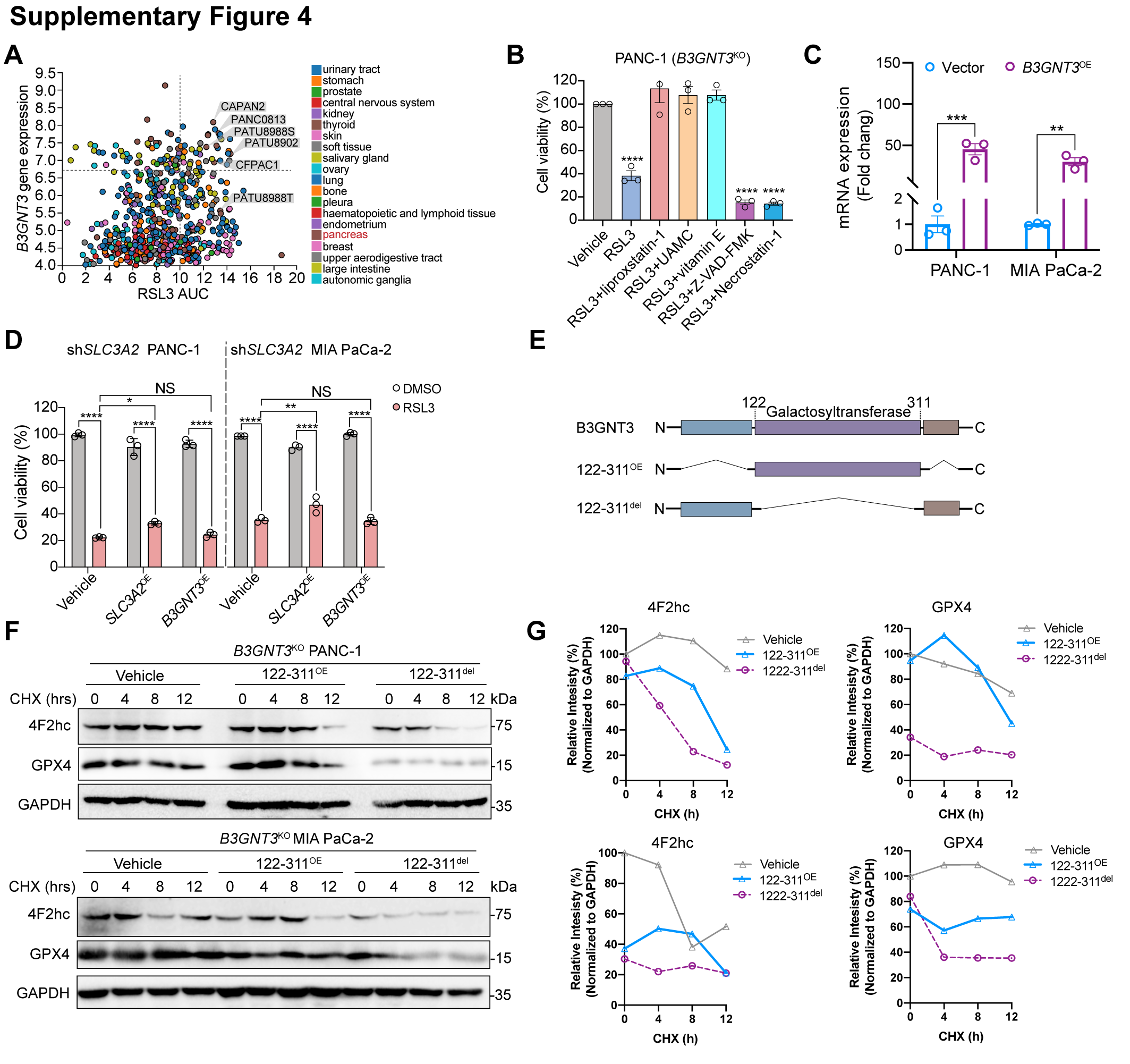

Supplement: Supplementary file 4 — Supplementary Figure 4 [file 41418_2023_1188_MOESM4_ESM.tif]

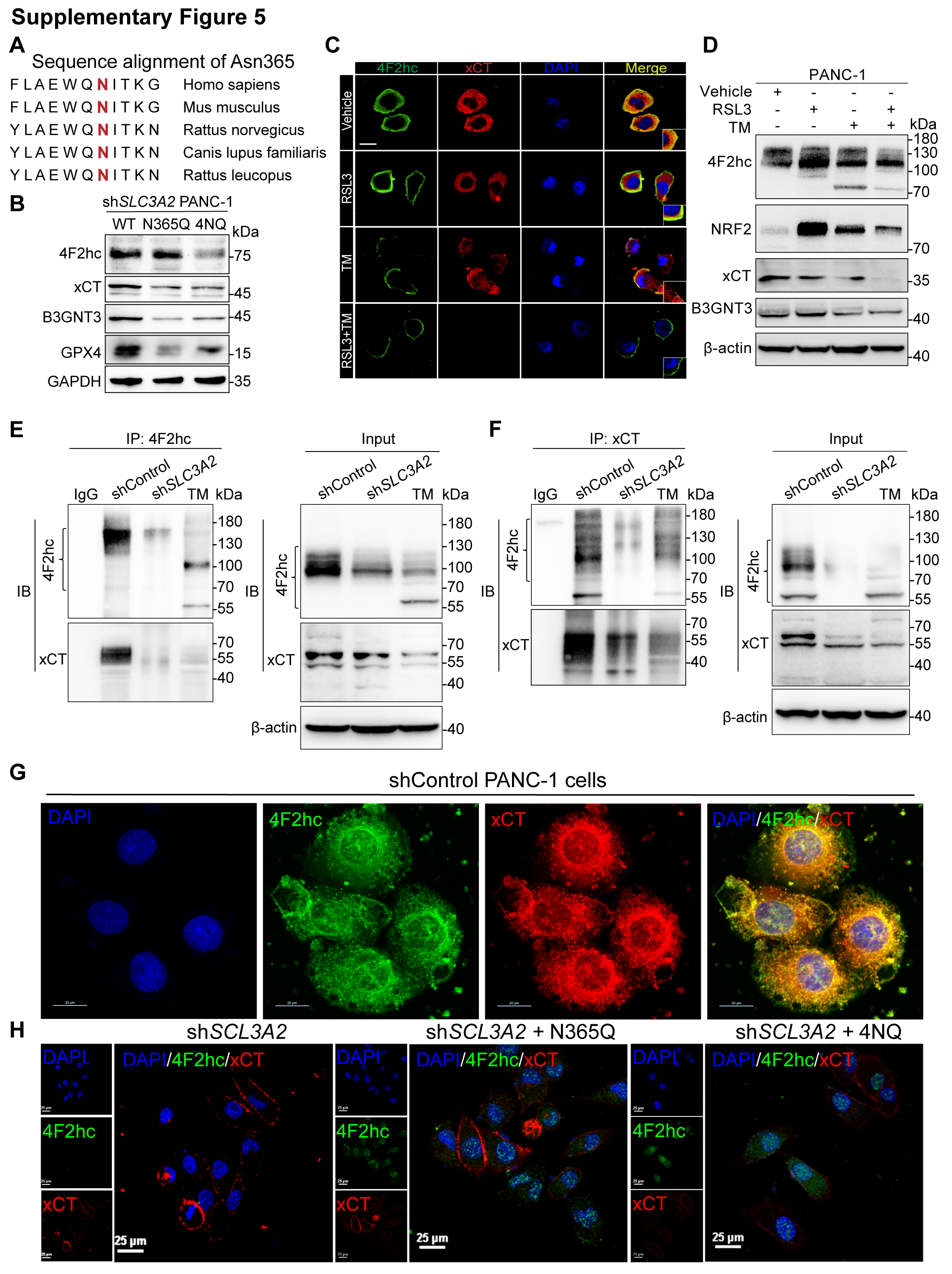

Supplement: Supplementary file 5 — Supplementary Figure 5 [file 41418_2023_1188_MOESM5_ESM.tif]

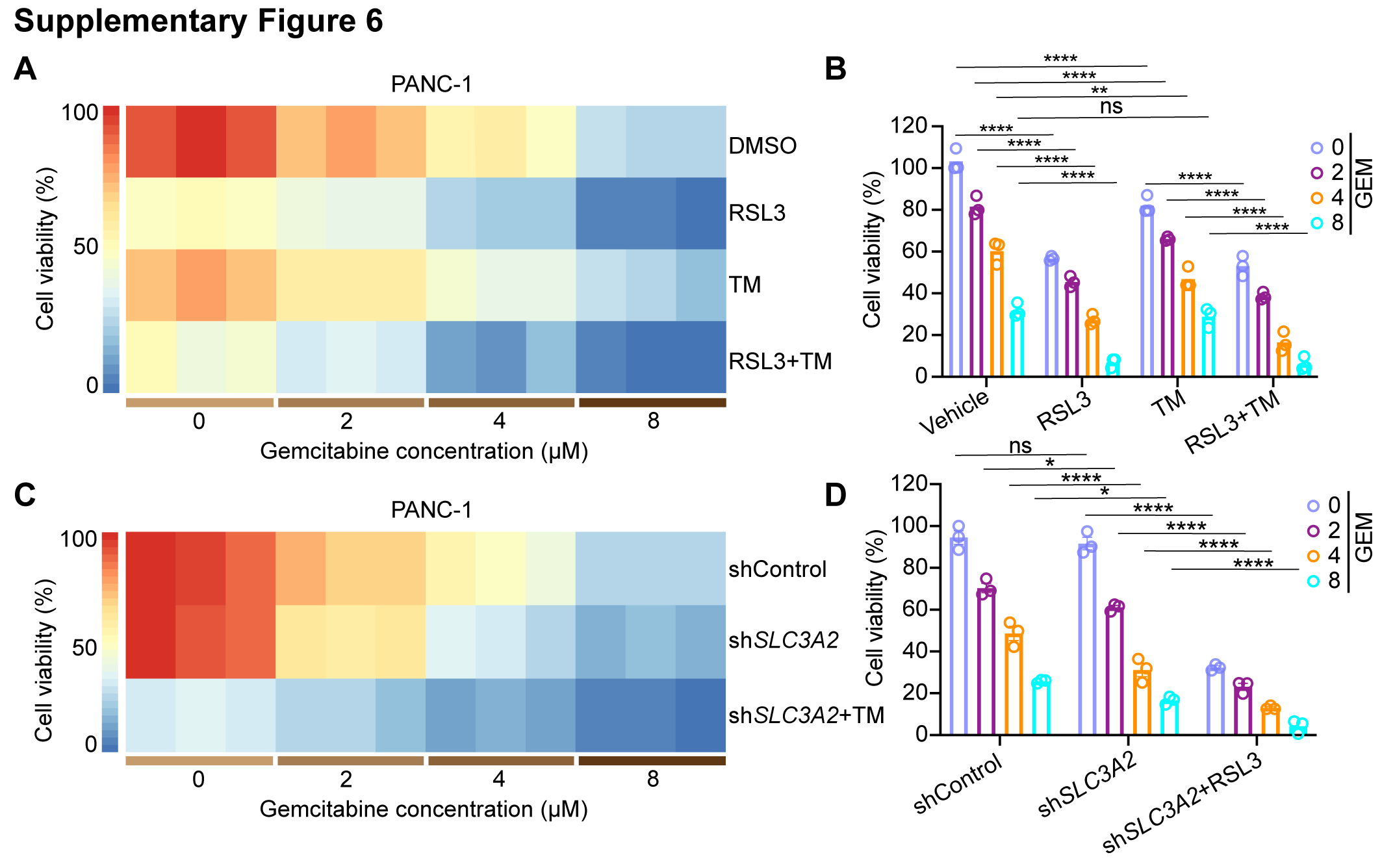

Supplement: Supplementary file 6 — Supplementary Figure 6 [file 41418_2023_1188_MOESM6_ESM.tif]

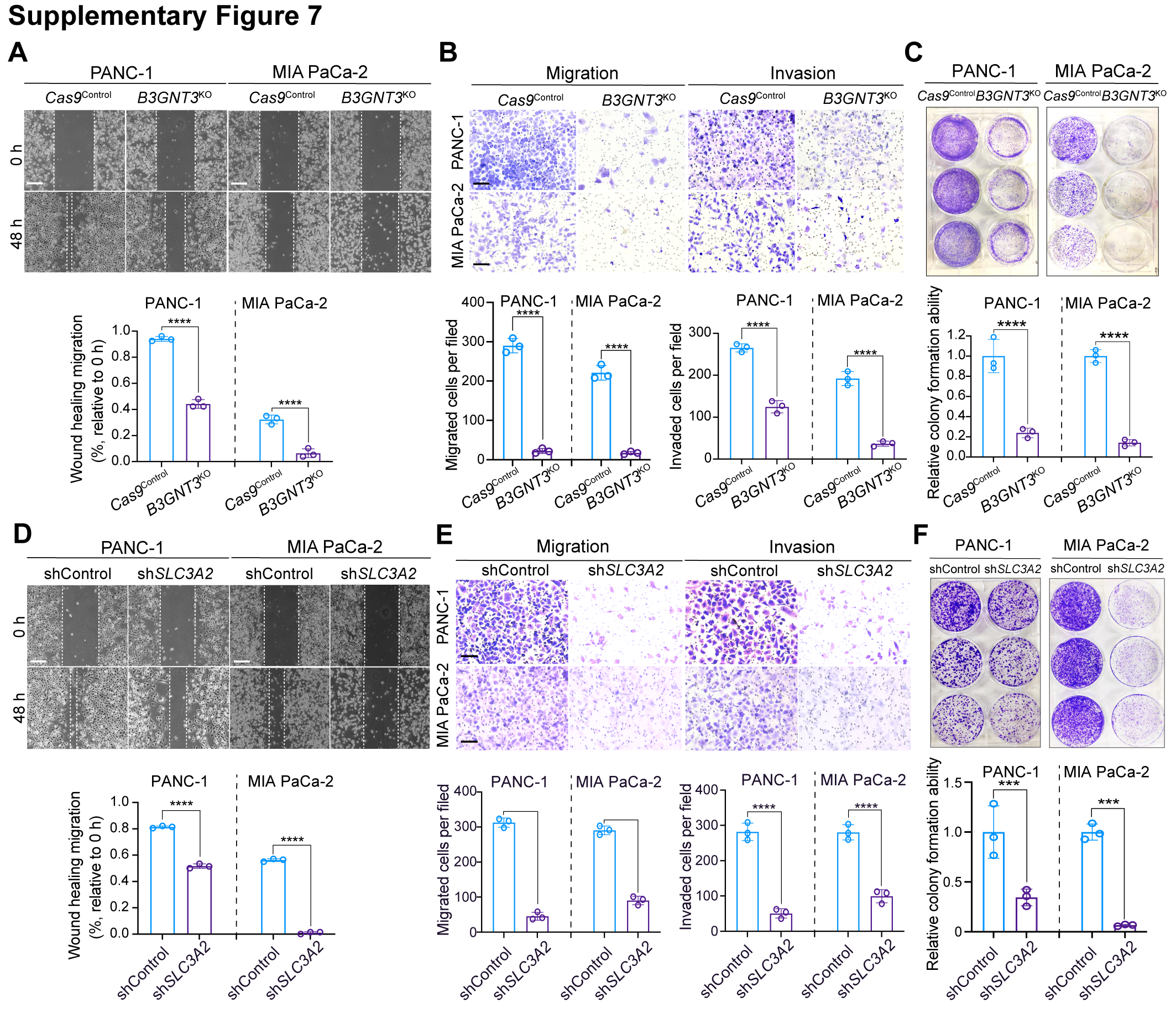

Supplement: Supplementary file 7 — Supplementary Figure 7 [file 41418_2023_1188_MOESM7_ESM.tif]

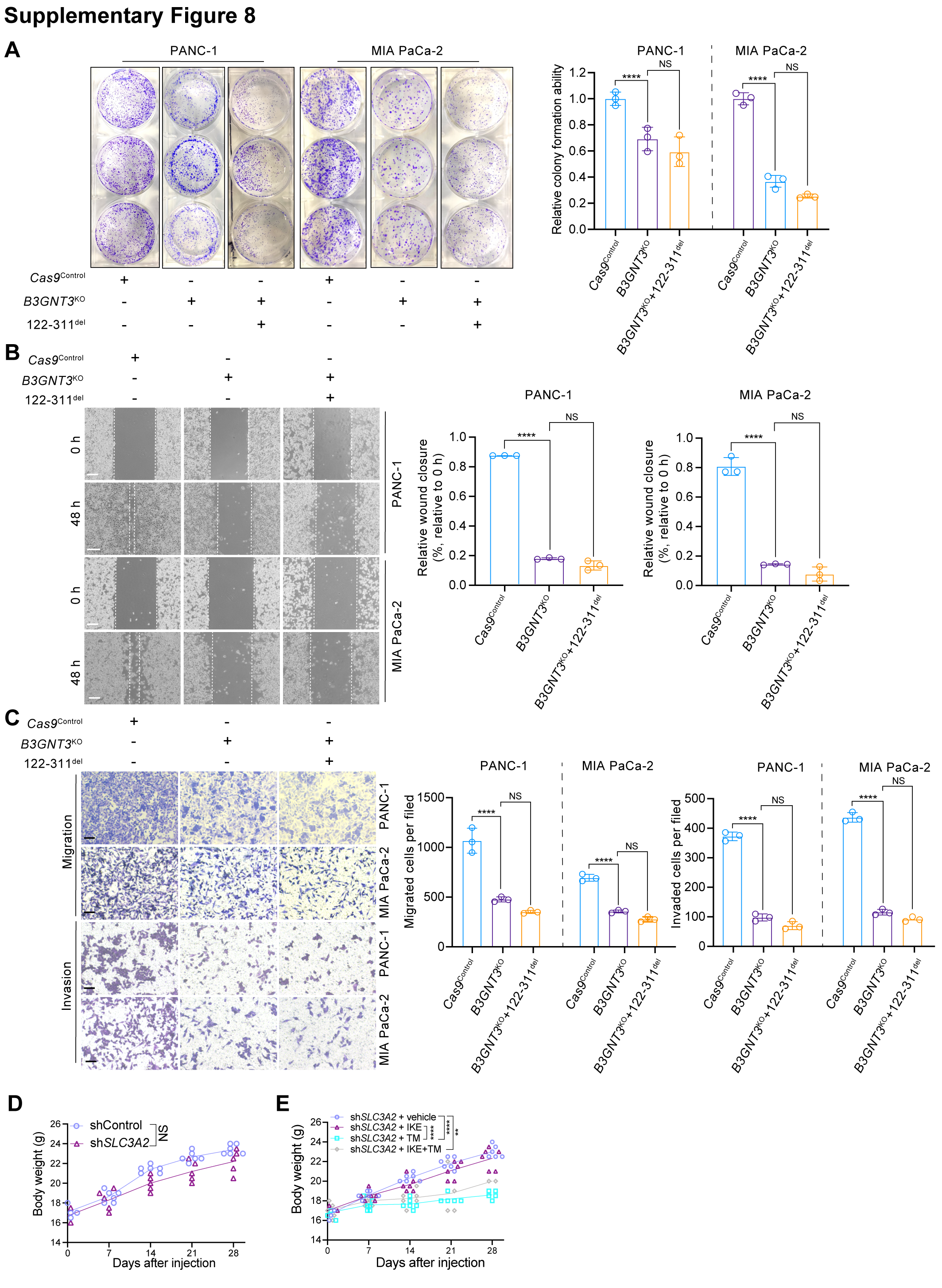

Supplement: Supplementary file 8 — Supplementary Figure 8 [file 41418_2023_1188_MOESM8_ESM.tif]

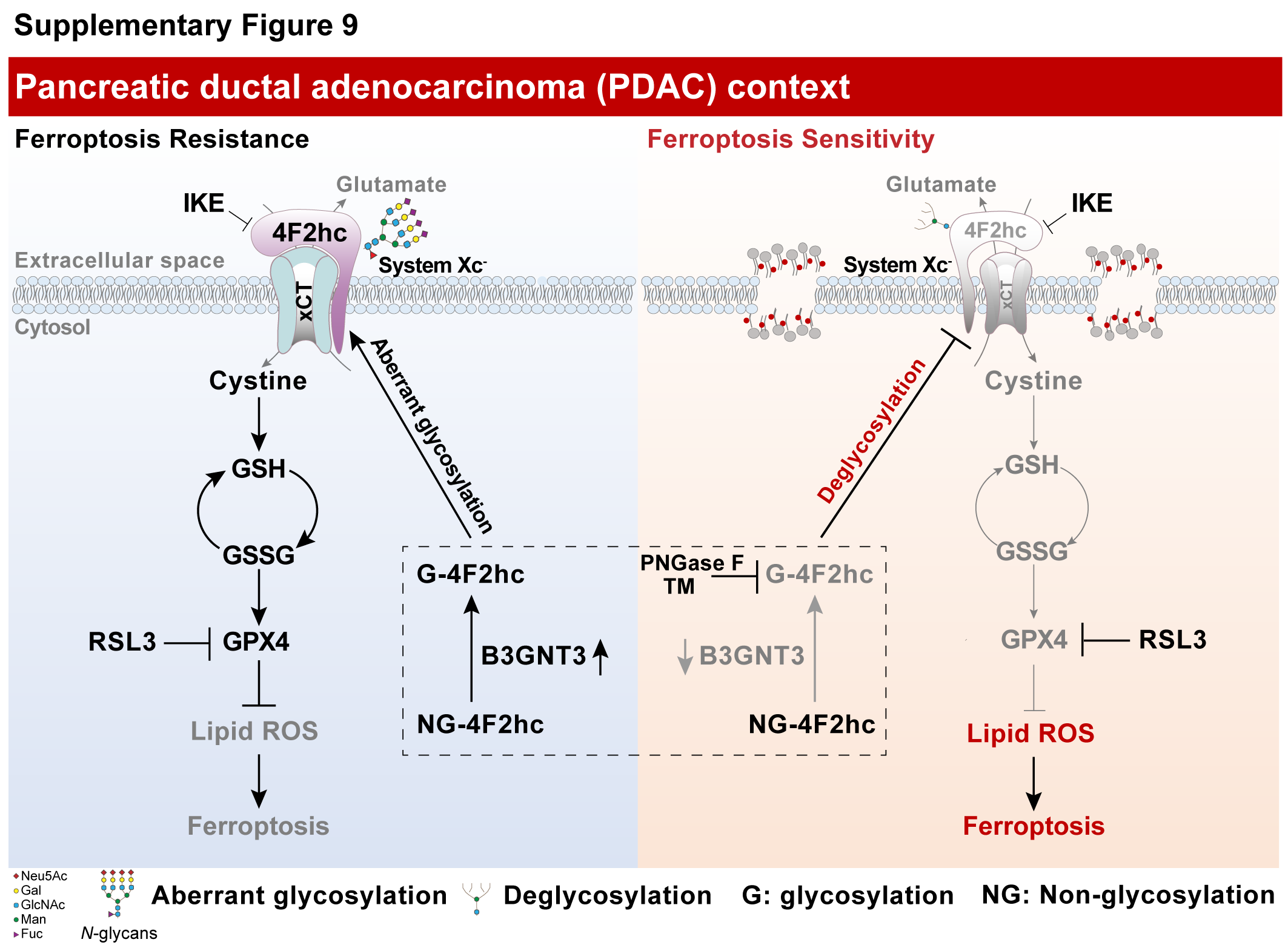

Supplement: Supplementary file 9 — Supplementary Figure 9 [file 41418_2023_1188_MOESM9_ESM.tif]
