# Supplementary material for: Targeting N-glycosylation of 4F2hc mediated by glycosyltransferase B3GNT3 sensitizes ferroptosis of pancreatic ductal adenocarcinoma
Source: Cell Death Differ. 2023 Jul 21;30(8):1988–2004. doi: 10.1038/s41418-023-01188-z (PMC10406883; doi:10.1038/s41418-023-01188-z)
Supplement: Supplementary file 10 — Supplementary Table 1 [file 41418_2023_1188_MOESM10_ESM.docx]

**Supplementary Table 1.** The Data sets of ferroptosis-related genes (FRGs) and glycosyltransferases genes.

| **Ferroptosis related genes** | | |  | **Differential N+O Glycoproteins** |  |
| --- | --- | --- | --- | --- | --- |
| Suppressor(208) | Driver(255) | Marker(125) | Intesection(387) | DNGPs (200) | DOGPs (23) |
| SLC7A11 | RPL8 | PTGS2 | ABCC1 | 2A5D | AMPN |
| GPX4 | IREB2 | DUSP1 | ABHD12 | 5NTD | CATB |
| AKR1C1 | ATP5MC3 | NOS2 | ACADSB | ADA17 | CATD |
| AKR1C2 | CS | NCF2 | ACO1 | AGAL | CD10 |
| AKR1C3 | EMC2 | MT3 | ACOT1 | AGRE5 | CD15 |
| GPX4 | ACSF2 | UBC | ACSF2 | AGRG6 | CD47 |
| RB1 | NOX1 | ALB | ACSL3 | AMPN | CD99 |
| HSPB1 | CYBB | TXNRD1 | ACSL4 | ANO6 | CDCP |
| HSF1 | NOX3 | SRXN1 | ACVR1B | APLP2 | CENP |
| SLC7A11 | NOX4 | GPX2 | AEBP2 | APMAP | CNG1 |
| GPX4 | NOX5 | BNIP3 | AGPAT3 | ASAH1 | CO1A |
| GCLC | DUOX1 | OXSR1 | AGPS | AT1B1 | FKB1 |
| SLC7A11 | DUOX2 | SELENOS | AHCY | AT1B3 | ITB1 |
| NFE2L2 | G6PD | ANGPTL7 | AIFM2 | ATRN | LAMP2 |
| SQSTM1 | PGD | CHAC1 | AKR1C1 | B3GLT | LMAN |
| NQO1 | VDAC2 | SLC7A11 | AKR1C3 | BASI | MPRI |
| HMOX1 | PIK3CA | DDIT4 | ALB | BSCL2 | NICA |
| FTH1 | FLT3 | LOC284561 | ALDH3A2 | BTD | NPC1 |
| MUC1 | SCP2 | ASNS | ALOX12 | CAHD1 | PICA |
| SLC3A2 | TP53 | TSC22D3 | ALOX12B | CATB | RCN1 |
| MT1G | ACSL4 | DDIT3 | ALOX15 | CATC | SAP |
| NFE2L2 | LPCAT3 | JDP2 | ALOX15B | CATD | SUN1 |
| SLC40A1 | NRAS | SESN2 | ALOX5 | CBPD | TMED |
| SLC7A11 | KRAS | SLC1A4 | ALOXE3 | CD109 |  |
| GPX4 | HRAS | PCK2 | AMN | CD151 |  |
| SLC7A11 | TF | TXNIP | ANGPTL7 | CD44 |  |
| CISD1 | TFRC | VLDLR | ANO6 | CD47 |  |
| SLC7A11 | TFR2 | GPT2 | AQP3 | CD59 |  |
| FANCD2 | SLC38A1 | PSAT1 | AQP5 | CD63 |  |
| GPX4 | SLC1A5 | LURAP1L | AQP8 | CD68 |  |
| NFE2L2 | GLS2 | SLC7A5 | AR | CD70 |  |
| FTMT | GOT1 | HERPUD1 | ARF6 | CDCP1 |  |
| HSPA5 | CARS1 | XBP1 | ARNTL | CEIP2 |  |
| ATF4 | TP53 | ATF3 | ARRDC3 | CERS2 |  |
| SLC7A11 | ALOX5 | SLC3A2 | ASNS | CLN5 |  |
| GPX4 | KEAP1 | CBS | ATF2 | CLP1L |  |
| GPX4 | HMOX1 | ATF4 | ATF3 | CLPT1 |  |
| HMOX1 | TP53 | ZNF419 | ATF4 | CLUS |  |
| ATF4 | TP53 | KLHL24 | ATG13 | CNTP1 |  |
| NFE2L2 | GLS2 | TRIB3 | ATG16L1 | CO1A1 |  |
| TP53 | ATG5 | ZFP69B | ATG3 | CO5A1 |  |
| SLC7A11 | ATG7 | ATP6V1G2 | ATG4D | CREG1 |  |
| HELLS | NCOA4 | VEGFA | ATG5 | CREL1 |  |
| SCD | TF | GDF15 | ATG7 | CRTAP |  |
| FADS2 | ALOX5 | TUBE1 | ATM | CXAR |  |
| SRC | ALOX12 | ARRDC3 | ATP5MC3 | D19L3 |  |
| STAT3 | ALOX12B | CEBPG | ATP6V1G2 | DNS2A |  |
| NFE2L2 | ALOX15 | SNORA16A | AURKA | DPP2 |  |
| PML | ALOX15B | RGS4 | BACH1 | ECE1 |  |
| MTOR | ALOXE3 | BLOC1S5-TXNDC5 | BAP1 | EGFR |  |
| NFS1 | PHKG2 | LOC390705 | BCAT2 | EMC1 |  |
| TP63 | TFRC | EIF2S1 | BECN1 | ENPL |  |
| SLC7A11 | ACO1 | KIM-1 | BID | EOGT |  |
| TP53 | IREB2 | IL6 | BLOC1S5-TXNDC5 | ERGI2 |  |
| CDKN1A | SLC38A1 | CXCL2 | BNIP3 | ERLN1 |  |
| MIR137 | GLS2 | RELA | BRD2 | ERMP1 |  |
| SLC40A1 | G6PDX | HSD17B11 | BRD3 | FAT1 |  |
| GPX4 | ULK1 | AGPAT3 | BRD4 | FKB10 |  |
| GPX4 | ATG3 | SETD1B | BRD7 | FKB14 |  |
| ENPP2 | ATG4D | HMOX1 | BRDT | FKBP9 |  |
| VDAC2 | ATG5 | TF | BRPF1 | FOXO3 |  |
| FH | BECN1 | FTL | CA9 | FUT11 |  |
| CISD2 | MAP1LC3A | RPL8 | CAPG | FXRD2 |  |
| SLC40A1 | GABARAPL2 | ATP5MC3 | CARS1 | G6PE |  |
| MIR9-1 | GABARAPL1 | TFRC | CAV1 | GALT1 |  |
| MIR9-2 | ATG16L1 | MAFG | CBS | GDF15 |  |
| MIR9-3 | WIPI1 | IL33 | CD44 | GGH |  |
| CBS | WIPI2 | FTH1 | CD82 | GGT7 |  |
| NFE2L2 | SNX4 | SLC40A1 | CDCA3 | GLCM |  |
| SQSTM1 | ATG13 | TF | CDH1 | GLT10 |  |
| GPX4 | ULK2 | TFRC | CDKN1A | GNS |  |
| ISCU | NCOA4 | FTH1 | CDKN2A | GSLG1 |  |
| FTH1 | ACSL4 | GPX4 | CDO1 | GT251 |  |
| ACSL3 | TP53 | HAMP | CEBPG | GXLT1 |  |
| OTUB1 | SAT1 | HSPB1 | CGAS | HEXA |  |
| CD44 | ALOX15 | NFE2L2 | CHAC1 | HGNAT |  |
| LINC00336 | ACSL4 | STEAP3 | CHMP5 | HPSE |  |
| STAT3 | LPCAT3 | DRD5 | CHMP6 | HS2ST |  |
| BRD4 | ALOX15 | GPX4 | CHP1 | HYOU1 |  |
| PRDX6 | ACSL4 | DRD4 | circ-TTBK2 | I17RB |  |
| MIR17 | KEAP1 | MAP3K5 | CircIL4R | ICAM1 |  |
| SCD | EGFR | MAPK14 | CISD1 | IGF1R |  |
| SESN2 | NOX4 | SLC2A1 | CISD2 | IKIP |  |
| NF2 | MAPK3 | SLC2A3 | CP | IL6RB |  |
| ARNTL | MAPK1 | SLC2A6 | CS | IMPA3 |  |
| HIF1A | BID | SLC2A8 | CTSB | IPRI |  |
| JUN | ACSL4 | SLC2A12 | CXCL2 | ITA2 |  |
| CA9 | ZEB1 | GLUT13 | CYB5R1 | ITA3 |  |
| HSPA5 | KEAP1 | SLC2A14 | CYBB | ITA5 |  |
| TMBIM4 | DPP4 | EIF2AK4 | CYP4F8 | ITA6 |  |
| HSPA5 | ALOX15 | EIF2S1 | DAZAP1 | ITAV |  |
| PLIN2 | ALOX12 | ATF4 | DCAF7 | ITB1 |  |
| MIR212 | CDKN2A | ALOX5 | DDIT3 | ITB3 |  |
| Fer1HCH | PEBP1 | ALOX12 | DDIT4 | ITB5 |  |
| AIFM2 | SOCS1 | ALOX15 | DECR1 | ITPR3 |  |
| AIFM2 | CDO1 | ALOX5 | DLD | L1CAM |  |
| LAMP2 | MYB | ACSF2 | DNAJB6 | LAMA5 |  |
| ZFP36 | HMOX1 | IREB2 | DPP4 | LAMB1 |  |
| GPX4 | MAPK8 | GPX4 | DRD4 | LAMB2 |  |
| PROM2 | MAPK9 | HMGB1 | DRD5 | LAMC1 |  |
| CHMP5 | MAPK1 | HMOX1 | DUOX1 | LAMP1 |  |
| CHMP6 | MAPK3 | NFE2L2 | DUOX2 | LAMP2 |  |
| AKR1C1 | SLC1A5 | ELAVL1 | DUSP1 | LCAP |  |
| AKR1C2 | CHAC1 | SLC3A2 | EGFR | LEMD2 |  |
| AKR1C3 | MAPK14 | SLC7A11 | EGLN2 | LGMN |  |
| CBS | LINC00472 | TFAP2C | EIF2AK4 | LICH |  |
| NFE2L2 | NOX4 | SP1 | EIF2S1 | LMA2L |  |
| CAV1 | GOT1 | HBA1 | ELAVL1 | LMAN2 |  |
| GCH1 | BECN1 | NNMT | ELOVL5 | LRP1 |  |
| SIRT3 | PRKAA2 | PLIN4 | EMC2 | LRP10 |  |
| DAZAP1 | PRKAA1 | HIC1 | ENPP2 | LTBP2 |  |
| PIR | ELAVL1 | STMN1 | EPAS1 | LYAG |  |
| GCLC | BAP1 | RRM2 | EPT1 | MBRL |  |
| FTL | TP53 | CAPG | FADS1 | MERTK |  |
| HCAR1 | ABCC1 | HNF4A | FADS2 | MFGM |  |
| SLC16A1 | ACSL4 | NGB | FANCD2 | MPRD |  |
| RRM2 | MIR6852 | YWHAE | FAR1 | MPRI |  |
| SCD | ACVR1B | GABPB1 | FBW7 | MPZL1 |  |
| NR4A1 | TGFBR1 | AURKA | FBXW7 | MRC2 |  |
| PIK3CA | BAP1 | MIR4715 | Fer1HCH | NAGAB |  |
| RPTOR | EPAS1 | RIPK1 | FH | NCEH1 |  |
| SREBF1 | HILPDA | PRDX1 | FLT3 | NCLN |  |
| SREBF2 | HIF1A | MIR30B | FNDC5 | NEUR1 |  |
| FZD7 | ALOX12 | MMP13 | FTH1 | NICA |  |
| NFE2L2 | ACSL4 | LRRFIP1 | FTL | NPC1 |  |
| NFE2L2 | HMOX1 |  | FTMT | NPTN |  |
| P4HB | IFNG |  | FXN | NRP1 |  |
| NT5DC2 | ANO6 |  | FZD7 | OSTM1 |  |
| BCAT2 | LPIN1 |  | G6PD | P2RX4 |  |
| HSF1 | HMGB1 |  | G6PDX | P3H1 |  |
| PLA2G6 | TNFAIP3 |  | GABARAPL1 | P4HA1 |  |
| MIR424 | TLR4 |  | GABARAPL2 | PCP |  |
| PARK7 | NOX4 |  | GABPB1 | PCYOX |  |
| FXN | ATF3 |  | GCH1 | PGLT1 |  |
| SUV39H1 | ATM |  | GCLC | PLBL1 |  |
| ATF2 | YY1AP1 |  | GDF15 | PLBL2 |  |
| CDKN1A | EGLN2 |  | GLRX5 | PLGT3 |  |
| FTH1 | MIOX |  | GLS2 | PLOD1 |  |
| NFE2L2 | TAZ |  | GLUT13 | PLOD2 |  |
| STAT3 | MTDH |  | GOT1 | PLOD3 |  |
| ACOT1 | IDH1 |  | GPAT4 | PLXB2 |  |
| NFE2L2 | SIRT1 |  | GPT2 | PO210 |  |
| ALDH3A2 | TAZ |  | GPX2 | POMT |  |
| NFE2L2 | BECN1 |  | GPX4 | POMT1 |  |
| STK11 | FBXW7 |  | GRIA3 | POMT2 |  |
| FNDC5 | PANX1 |  | GSK3B | PON2 |  |
| CircIL4R | DNAJB6 |  | HAMP | PPAL |  |
| CDH1 | BACH1 |  | HBA1 | PPGB |  |
| NFE2L2 | ACSL4 |  | HCAR1 | PPT1 |  |
| MIR214 | LONP1 |  | HDDC3 | PRS23 |  |
| NEDD4L | CD82 |  | HELLS | PTPRJ |  |
| SQSTM1 | IL1B |  | HERPUD1 | PTTG |  |
| TF | CTSB |  | HIC1 | QSOX1 |  |
| FTMT | POR |  | HIF1A | QSOX2 |  |
| BRD2 | CYB5R1 |  | HILPDA | RCN1 |  |
| BRD3 | ELOVL5 |  | HMGB1 | RCN3 |  |
| BRD4 | FADS1 |  | HMOX1 | S12A4 |  |
| BRDT | ALOX12 |  | HNF4A | S12A6 |  |
| SCD | FBW7 |  | HRAS | S12A7 |  |
| SLC7A11 | PTEN |  | hsa_circ_0008367 | S39AE |  |
| DECR1 | NR1D1 |  | HSD17B11 | S4A7 |  |
| NFE2L2 | NR1D2 |  | HSF1 | SAP |  |
| GPX4 | TBK1 |  | HSPA5 | SCRB2 |  |
| SLC7A11 | IL6 |  | HSPB1 | SE1L1 |  |
| NFE2L2 | USP7 |  | IDH1 | SEM3C |  |
| GLRX5 | miR-182-5p |  | IDH2 | SEM4B |  |
| GPX4 | miR-378a-3p |  | IFNG | SERPH |  |
| NCOA3 | CTSB |  | IL1B | SLC3A2 |  |
| NR5A2 | ACSL4 |  | IL33 | SORT |  |
| GPX4 | ATF4 |  | IL6 | SPIT2 |  |
| MTOR | BECN1 |  | INTS2 | SPP2A |  |
| PANX2 | AQP3 |  | IREB2 | SSRB |  |
| RHEBP1 | AQP5 |  | ISCU | STT3A |  |
| TFAP2A | AQP8 |  | JDP2 | STT3B |  |
| CP | LINC00618 |  | JUN | SUN1 |  |
| SLC7A11 | IREB2 |  | KDM3B | SYPL1 |  |
| ARF6 | MT1DP |  | KEAP1 | T106B |  |
| GDF15 | ACSL4 |  | KIM-1 | TECT3 |  |
| ABHD12 | PEX10 |  | KLHL24 | TFRC |  |
| PPP1R13L | KEAP1 |  | KRAS | TGFB2 |  |
| TFAM | AGPAT3 |  | LAMP2 | TGON2 |  |
| KDM3B | PEX12 |  | LCE2C | THY1 |  |
| RNF113A | CHP1 |  | LIG3 | TIMP1 |  |
| PARK7 | GPAT4 |  | LINC00336 | TM131 |  |
| AHCY | BRPF1 |  | LINC00472 | TM245 |  |
| FXN | OSBPL9 |  | LINC00618 | TM2D1 |  |
| circ-TTBK2 | INTS2 |  | LOC284561 | TM2D3 |  |
| MIR522 | MMD |  | LOC390705 | TM87A |  |
| IDH2 | CYP4F8 |  | LONP1 | TM87B |  |
| PPARA | MLLT1 |  | LPCAT3 | TMED4 |  |
| NOS2 | TTPA |  | LPIN1 | TMED9 |  |
| SIAH2 | GRIA3 |  | LRRFIP1 | TMM43 |  |
| RELA | EPT1 |  | LURAP1L | TMX3 |  |
| PRKAA2 | POM121L12 |  | LYRM1 | TNR5 |  |
| VDR | LIG3 |  | MAFG | TPBG |  |
| NEDD4 | AEBP2 |  | MAP1LC3A | TSN15 |  |
| FXN | AGPS |  | MAP3K11 | UGGG2 |  |
| AIFM2 | CDCA3 |  | MAP3K5 | UROK |  |
| PRDX1 | PEX2 |  | MAPK1 | VAS1 |  |
| AR | LPCAT3 |  | MAPK14 | VTNC |  |
| CBS | PEX6 |  | MAPK3 |  |  |
| NFE2L2 | TIMM9 |  | MAPK8 |  |  |
| CHMP5 | DCAF7 |  | MAPK9 |  |  |
| CHMP6 | LCE2C |  | MDM2 |  |  |
| HMOX1 | FAR1 |  | MDM4 |  |  |
| ZFP36 | PHF21A |  | MFN2 |  |  |
| LAMP2 | SMAD7 |  | MIOX |  |  |
| MTF1 | LYRM1 |  | miR-182-5p |  |  |
|  | AMN |  | miR-378a-3p |  |  |
|  | PEX3 |  | MIR137 |  |  |
|  | MTCH1 |  | MIR17 |  |  |
|  | ZEB1 |  | MIR212 |  |  |
|  | SIRT1 |  | MIR214 |  |  |
|  | ACADSB |  | MIR30B |  |  |
|  | PVT1 |  | MIR424 |  |  |
|  | hsa_circ_0008367 |  | MIR4715 |  |  |
|  | SLC39A14 |  | MIR522 |  |  |
|  | NCOA4 |  | MIR6852 |  |  |
|  | MAP3K11 |  | MIR761 |  |  |
|  | GSK3B |  | MIR9-1 |  |  |
|  | MAPK8 |  | MIR9-2 |  |  |
|  | BRD7 |  | MIR9-3 |  |  |
|  | TP53 |  | MLLT1 |  |  |
|  | SLC25A28 |  | MMD |  |  |
|  | ACSL4 |  | MMP13 |  |  |
|  | MFN2 |  | MT1DP |  |  |
|  | ACSL4 |  | MT1G |  |  |
|  | SLC11A2 |  | MT3 |  |  |
|  | ZFAS1 |  | MTCH1 |  |  |
|  | SLC38A1 |  | MTDH |  |  |
|  | TSC1 |  | MTF1 |  |  |
|  | PEBP1 |  | MTOR |  |  |
|  | TGFB1 |  | MUC1 |  |  |
|  | SNCA |  | MYB |  |  |
|  | SIRT3 |  | NCF2 |  |  |
|  | PRKAA2 |  | NCOA3 |  |  |
|  | TFRC |  | NCOA4 |  |  |
|  | CGAS |  | NEDD4 |  |  |
|  | STING1 |  | NEDD4L |  |  |
|  | HDDC3 |  | NF2 |  |  |
|  | MIR761 |  | NFE2L2 |  |  |
|  | MDM2 |  | NFS1 |  |  |
|  | MDM4 |  | NGB |  |  |
|  | ALOX15 |  | NNMT |  |  |
|  | POR |  | NOS2 |  |  |
|  | MIR214 |  | NOX1 |  |  |
|  | DLD |  | NOX3 |  |  |
|  | LONP1 |  | NOX4 |  |  |
|  | ACSL4 |  | NOX5 |  |  |
|  | BACH1 |  | NQO1 |  |  |
|  | DNAJB6 |  | NR1D1 |  |  |
|  | WWTR1 |  | NR1D2 |  |  |
|  | SIRT1 |  | NR4A1 |  |  |
|  | ATM |  | NR5A2 |  |  |
|  | PRKCA |  | NRAS |  |  |
|  |  |  | NT5DC2 |  |  |
|  |  |  | OSBPL9 |  |  |
|  |  |  | OTUB1 |  |  |
|  |  |  | OXSR1 |  |  |
|  |  |  | P4HB |  |  |
|  |  |  | PANX1 |  |  |
|  |  |  | PANX2 |  |  |
|  |  |  | PARK7 |  |  |
|  |  |  | PCK2 |  |  |
|  |  |  | PEBP1 |  |  |
|  |  |  | PEX10 |  |  |
|  |  |  | PEX12 |  |  |
|  |  |  | PEX2 |  |  |
|  |  |  | PEX3 |  |  |
|  |  |  | PEX6 |  |  |
|  |  |  | PGD |  |  |
|  |  |  | PHF21A |  |  |
|  |  |  | PHKG2 |  |  |
|  |  |  | PIK3CA |  |  |
|  |  |  | PIR |  |  |
|  |  |  | PLA2G6 |  |  |
|  |  |  | PLIN2 |  |  |
|  |  |  | PLIN4 |  |  |
|  |  |  | PML |  |  |
|  |  |  | POM121L12 |  |  |
|  |  |  | POR |  |  |
|  |  |  | PPARA |  |  |
|  |  |  | PPP1R13L |  |  |
|  |  |  | PRDX1 |  |  |
|  |  |  | PRDX6 |  |  |
|  |  |  | PRKAA1 |  |  |
|  |  |  | PRKAA2 |  |  |
|  |  |  | PRKCA |  |  |
|  |  |  | PROM2 |  |  |
|  |  |  | PSAT1 |  |  |
|  |  |  | PTEN |  |  |
|  |  |  | PTGS2 |  |  |
|  |  |  | PVT1 |  |  |
|  |  |  | RB1 |  |  |
|  |  |  | RELA |  |  |
|  |  |  | RGS4 |  |  |
|  |  |  | RHEBP1 |  |  |
|  |  |  | RIPK1 |  |  |
|  |  |  | RNF113A |  |  |
|  |  |  | RPL8 |  |  |
|  |  |  | RPTOR |  |  |
|  |  |  | RRM2 |  |  |
|  |  |  | SAT1 |  |  |
|  |  |  | SCD |  |  |
|  |  |  | SCP2 |  |  |
|  |  |  | SELENOS |  |  |
|  |  |  | SESN2 |  |  |
|  |  |  | SETD1B |  |  |
|  |  |  | SIAH2 |  |  |
|  |  |  | SIRT1 |  |  |
|  |  |  | SIRT3 |  |  |
|  |  |  | SLC11A2 |  |  |
|  |  |  | SLC16A1 |  |  |
|  |  |  | SLC1A4 |  |  |
|  |  |  | SLC1A5 |  |  |
|  |  |  | SLC25A28 |  |  |
|  |  |  | SLC2A1 |  |  |
|  |  |  | SLC2A12 |  |  |
|  |  |  | SLC2A14 |  |  |
|  |  |  | SLC2A3 |  |  |
|  |  |  | SLC2A6 |  |  |
|  |  |  | SLC2A8 |  |  |
|  |  |  | SLC38A1 |  |  |
|  |  |  | SLC39A14 |  |  |
|  |  |  | SLC3A2 |  |  |
|  |  |  | SLC40A1 |  |  |
|  |  |  | SLC7A11 |  |  |
|  |  |  | SLC7A5 |  |  |
|  |  |  | SMAD7 |  |  |
|  |  |  | SNCA |  |  |
|  |  |  | SNORA16A |  |  |
|  |  |  | SNX4 |  |  |
|  |  |  | SOCS1 |  |  |
|  |  |  | SP1 |  |  |
|  |  |  | SQSTM1 |  |  |
|  |  |  | SRC |  |  |
|  |  |  | SREBF1 |  |  |
|  |  |  | SREBF2 |  |  |
|  |  |  | SRXN1 |  |  |
|  |  |  | STAT3 |  |  |
|  |  |  | STEAP3 |  |  |
|  |  |  | STING1 |  |  |
|  |  |  | STK11 |  |  |
|  |  |  | STMN1 |  |  |
|  |  |  | SUV39H1 |  |  |
|  |  |  | TAZ |  |  |
|  |  |  | TBK1 |  |  |
|  |  |  | TF |  |  |
|  |  |  | TFAM |  |  |
|  |  |  | TFAP2A |  |  |
|  |  |  | TFAP2C |  |  |
|  |  |  | TFR2 |  |  |
|  |  |  | TFRC |  |  |
|  |  |  | TGFB1 |  |  |
|  |  |  | TGFBR1 |  |  |
|  |  |  | TIMM9 |  |  |
|  |  |  | TLR4 |  |  |
|  |  |  | TMBIM4 |  |  |
|  |  |  | TNFAIP3 |  |  |
|  |  |  | TP53 |  |  |
|  |  |  | TP63 |  |  |
|  |  |  | TRIB3 |  |  |
|  |  |  | TSC1 |  |  |
|  |  |  | TSC22D3 |  |  |
|  |  |  | TTPA |  |  |
|  |  |  | TUBE1 |  |  |
|  |  |  | TXNIP |  |  |
|  |  |  | TXNRD1 |  |  |
|  |  |  | UBC |  |  |
|  |  |  | ULK1 |  |  |
|  |  |  | ULK2 |  |  |
|  |  |  | USP7 |  |  |
|  |  |  | VDAC2 |  |  |
|  |  |  | VDR |  |  |
|  |  |  | VEGFA |  |  |
|  |  |  | VLDLR |  |  |
|  |  |  | WIPI1 |  |  |
|  |  |  | WIPI2 |  |  |
|  |  |  | WWTR1 |  |  |
|  |  |  | XBP1 |  |  |
|  |  |  | YWHAE |  |  |
|  |  |  | YY1AP1 |  |  |
|  |  |  | ZEB1 |  |  |
|  |  |  | ZFAS1 |  |  |
|  |  |  | ZFP36 |  |  |
|  |  |  | ZFP69B |  |  |
|  |  |  | ZNF419 |  |  |
