# Supplementary material for: Targeting N-glycosylation of 4F2hc mediated by glycosyltransferase B3GNT3 sensitizes ferroptosis of pancreatic ductal adenocarcinoma
Source: Cell Death Differ. 2023 Jul 21;30(8):1988–2004. doi: 10.1038/s41418-023-01188-z (PMC10406883; doi:10.1038/s41418-023-01188-z)
Supplement: Supplementary file 11 — Supplementary Table 2 [file 41418_2023_1188_MOESM11_ESM.docx]

**Supplementary Table 2.** The sequences of the primers for qRT-PCR were used in this study.

| **Quantitative Reverse Transcription PCR primer sequences** | | | |
| --- | --- | --- | --- |
| Species | Gene | Primer | 5' to 3' |
| Human | SLC7A11 | F | TCCTGCTTTGGCTCCATGAACG |
|  |  | R | AGAGGAGTGCTTGCGGACAT |
|  | B3GNT3 | F | TCTTCAACCTCACGCTCAAG |
|  |  | R | GTGTGCAAAGACGTCATCATC |
|  | SLC3A2 | F | CTGGTGCCGTGGTCATAATC |
|  |  | R | GCTCAGGTAATCGAGACGCC |
|  | NFE2L2 | F | ACACGGTCCACAGCTCATC |
|  |  | R | TGTCAATCAAATCCATGTCCTG |
|  | GPX4 | F | ACAAGAACGGCTGCGTGGTGAA |
|  |  | R | GCCACACACTTGTGGAGCTAGA |
|  | DHODH | F | CCACGGGAGATGAGCGTTTC |
|  |  | R | CAGGGAGGTGAAGCGAACA |
|  | ACSL4 | F | GCTATCTCCTCAGACACACCGA |
|  |  | R | AGGTGCTCCAACTCTGCCAGTA |
|  | B3GNT5 | F | GGGCCTCGCTACCAATACTTG |
|  |  | R | CGGAACGTCGATCATAGTTTTCA |
|  | GCNT4 | F | GTTGTGGCAATGACCAGTGAT |
|  |  | R | AGCATGGAAAAGCCTTTCAACC |
|  | ASGR1 | F | AAGATGAAGTCGCTAGAGTCCC |
|  |  | R | CAGGTCAGACAGAACTGCTT |
|  | GAPDH | F | CCCCTTCATTGACCTCAAC |
|  |  | R | CAAAGTTGTCATGGATGACC |
|  |  |  |  |
| **sgRNA targets sequences** | | | |
| B3GNT3 sgRNA | | GGATGAAGTATCTCCGGCAC | |
|  |  |  |  |
| **shRNA targets sequences** | | | |
| nontargeting sequence | | TTCTCCGAACGTGTCACGT | |
| SLC3A2-sh#1 | | TGGGTCCAATTCACAAGAA | |
| SLC3A2-sh#2 | | AGAAGAATGGTCTGGTGAA | |
| SLC3A2-sh#3 | | CGTGTCATTCTGGACCTTA | |
|  |  |  |  |
| **OE-B3GNT3 targets sequences** | | | |
| Species | Gene | Primer | 5' to 3' |
| human | B3GNT3 | F | CGCAAATGGGCGGTAGGCGTG |
|  |  | R | CCTCTACAAATGTGGTATGGC |
|  |  |  |  |
| **siB3GNT3 targets sequences** | | | |
| siB3GNT3-#1 | | CGCAGCACGTTCAGAACTT | |
| siB3GNT3-#2 | | TGCACCGCTTCCTACCTTA | |
| siB3GNT3-#3 | | GGATGATGACGTCTTTGCA | |
