# Supplementary material for: Targeting N-glycosylation of 4F2hc mediated by glycosyltransferase B3GNT3 sensitizes ferroptosis of pancreatic ductal adenocarcinoma
Source: Cell Death Differ. 2023 Jul 21;30(8):1988–2004. doi: 10.1038/s41418-023-01188-z (PMC10406883; doi:10.1038/s41418-023-01188-z)
Supplement: Supplementary file 12 — Supplementary Table 3 [file 41418_2023_1188_MOESM12_ESM.docx]

**Supplementary Table 3. Association of clinicopathological features with B3GNT3 and 4F2hc protein expression.**

| Variables | N | B3GNT3 | | | | N | 4F2hc | | |
| --- | --- | --- | --- | --- | --- | --- | --- | --- | --- |
|  |  | Low | High | *P* value | |  | Low | High | *P* value |
| Sex |  |  |  | | 0.149 |  |  |  | 0.774 |
| Female | 119 | 62 (52.1) | 57 (47.9) | |  | 110 | 43 (39.1) | 67 (60.9) |  |
| Male | 137 | 59 (43.1) | 78 (56.9) | |  | 132 | 54 (40.9) | 78 (59.1) |  |
| Age, years |  |  |  | | 0.903 |  |  |  | 0.649 |
| <60 | 109 | 52 (47.7) | 57 (52.3) | |  | 103 | 43 (41.7) | 60 (58.3) |  |
| ≧60 | 147 | 69 (46.9) | 78 (53.1) | |  | 139 | 54 (38.8) | 85 (61.2) |  |
| Location |  |  |  | | 0.359 |  |  |  | 0.399 |
| Head | 164 | 74 (45.1) | 90 (54.9) | |  | 150 | 57 (38.0) | 93 (62.0) |  |
| Body & neck | 92 | 47 (51.1) | 45 (48.9) | |  | 92 | 40 (43.5) | 52 (56.5) |  |
| LVI | |  |  | | 0.872 |  |  |  | 0.467 |
| Absent | 160 | 75 (46.9) | 85 (53.1) | |  | 153 | 64 (41.8) | 89 (58.2) |  |
| Present | 96 | 46 (47.9) | 50 (52.1) | |  | 89 | 33 (37.1) | 56 (62.9) |  |
| PNI |  |  |  | | 0.301 |  |  |  | 0.377 |
| Absent | 89 | 46 (51.7) | 43 (48.3) | |  | 77 | 34 (44.2) | 43 (55.8) |  |
| Present | 167 | 75 (44.9) | 92 (55.1) | |  | 165 | 63 (38.2) | 102 (61.8) |  |
| Tumor stage |  |  |  | | 0.001 |  |  |  | 0.277 |
| T1-2 | 192 | 102 (53.1) | 90 (46.9) | |  | 178 | 75 (42.1) | 103 (57.9) |  |
| T3 | 64 | 19 (29.7) | 45 (70.3) | |  | 64 | 22 (34.4) | 42 (65.6) |  |
| Node stage | |  |  | | 0.160 |  |  |  | 0.473 |
| N0-1 | 211 | 104 (49.3) | 107 (50.7) | |  | 202 | 83 (41.1) | 119 (58.9) |  |
| N2 | 45 | 17 (37.8) | 28 (62.2) | |  | 40 | 14 (35.0) | 26 (65.0) |  |
| Distant metastasis |  |  |  | | 0.287 |  |  |  | 0.149 |
| M0 | 248 | 119 (48.0) | 129 (52.0) | |  | 234 | 96 (41.0) | 138 (59.0) |  |
| M1 | 8 | 2 (25.0) | 6 (75.0) | |  | 8 | 1 (12.5) | 7 (87.5) |  |
| Tumor differentiation |  |  |  | | 0.218 |  |  |  | <0.001 |
| Well & moderately differentiated | 157 | 79 (50.3) | 78 (49.7) | |  | 155 | 75 (48.4) | 80 (51.6) |  |
| Poorly differentiated | 99 | 42 (42.4) | 57 (57.6) | |  | 87 | 22 (25.3) | 65 (74.7) |  |
| AJCC |  |  |  | | 0.033 |  |  |  | 0.368 |
| Ⅰ-Ⅱ | 201 | 102 (50.7) | 99 (49.3) | |  | 192 | 82 (42.7) | 110 (57.3) |  |
| Ⅲ-Ⅳ | 55 | 19 (34.5) | 36 (65.5) | |  | 40 | 14 (35.0) | 26 (65.0) |  |

AJCC, American Joint Committee on Cancer; LVI, lymphovascular invasion; PNI, perineural invasion.
