# Supplementary material for: Targeting N-glycosylation of 4F2hc mediated by glycosyltransferase B3GNT3 sensitizes ferroptosis of pancreatic ductal adenocarcinoma
Source: Cell Death Differ. 2023 Jul 21;30(8):1988–2004. doi: 10.1038/s41418-023-01188-z (PMC10406883; doi:10.1038/s41418-023-01188-z)
Supplement: Supplementary file 13 — Supplementary Table 4 [file 41418_2023_1188_MOESM13_ESM.docx]

**Supplementary Table 4.** Univariate analysis of factors potentially associated with progression-free survival and disease-specific survival.

|  | Progression-free survival | | Disease-specific survival | |
| --- | --- | --- | --- | --- |
|  | HR (95% CI) | P-value | HR (95% CI) | P-value |
| Sex |  | 0.014 |  | 0.056 |
| Female | 1 |  | 1 |  |
| Male | 1.419 (1.074–1.875) |  | 1.343 (0.992–1.818) |  |
| Age, years |  | 0.378 |  | 0.303 |
| <60 | 1 |  | 1 |  |
| ≥60 | 1.133 (0.859–1.494) |  | 1.173 (0.866–1.585) |  |
| Tumor differentiation |  | <0.001 |  | <0.001 |
| Moderately/well-differentiated | 1 |  | 1 |  |
| Poorly differentiated | 1.769 (1.339–2.338) |  | 2.035 (1.506–2.751) |  |
| Tumor stage |  | 0.001 |  | <0.001 |
| T1 | 1 |  | 1 |  |
| T2 | 1.658 (1.086–2.614) | 0.020 | 1.833 (1.109–3.031) | 0.018 |
| T3 | 2.439 (1.510–3.938) | <0.001 | 2.863 (1.671–4.903) | <0.001 |
| Node stage |  | <0.001 |  | <0.001 |
| N0 | 1 |  | 1 |  |
| N1 | 1.540 (1.130–2.100) | 0.006 | 1.588 (1.126–2.239) | 0.008 |
| N2 | 2.228 (1.500–3.310) | <0.001 | 2.343 (1.531–3.586) | <0.001 |
| Distant metastasis |  | 0.021 |  | 0.010 |
| M0 | 1 |  | 1 |  |
| M1 | 2.214 (1.130–4.338) |  | 2.436 (1.240–4.789) |  |
| AJCC stage |  | <0.001 |  | <0.001 |
| Ⅰ | 1 |  | 1 |  |
| Ⅱ | 1.816 (1.276–2.585) | 0.001 | 2.068 (1.374–3.111) | <0.001 |
| Ⅲ | 2.753 (1.780–4.257) | <0.001 | 3.187 (1.960–5.182) | <0.001 |
| Ⅳ | 4.561 (2.332–8.922) | <0.001 | 5.627 (2.797–11.318) | <0.001 |
| Adjuvant chemotherapy |  | 0.001 |  | <0.001 |
| No | 1 |  | 1 |  |
| Yes | 0.614 (0.459–0.823) |  | 0.507 (0.371–0.691) |  |
| B3GNT3 |  | <0.001 |  | <0.001 |
| Low | 1 |  | 1 |  |
| High | 1.990 (1.475–2.686) |  | 2.361 (1.685–3.308) |  |
| 4F2hc |  | <0.001 |  | <0.001 |
| Low | 1 |  | 1 |  |
| High | 2.425 (1.733–3.392) |  | 4.807 (3.066–7.538) |  |

AJCC, American Joint Committee on Cancer; CI, confidence interval; HR, hazard ratio.
