# Supplementary material for: Targeting N-glycosylation of 4F2hc mediated by glycosyltransferase B3GNT3 sensitizes ferroptosis of pancreatic ductal adenocarcinoma
Source: Cell Death Differ. 2023 Jul 21;30(8):1988–2004. doi: 10.1038/s41418-023-01188-z (PMC10406883; doi:10.1038/s41418-023-01188-z)
Supplement: Supplementary file 14 — Supplementary Table 5 [file 41418_2023_1188_MOESM14_ESM.docx]

**Supplementary Table 5.** Multivariate analysis of factors potentially associated with progression-free survival and disease-specific survival.

|  | Progression-free survival | | Disease-specific survival | | |
| --- | --- | --- | --- | --- | --- |
|  | HR (95% CI) | P-value | HR (95% CI) | P-value | |
| Tumor differentiation |  | 0.050 |  | 0.054 | |
| Moderately/well-differentiated | 1 |  | 1 |  |  |
| Poorly differentiated | 1.395 (1.000–1.944) |  | 1.421 (0.994–2.033) |  | |
| AJCC stage |  | 0.001 |  | 0.001 | |
| Ⅰ | 1 |  | 1 |  | |
| Ⅱ | 1.802 (1.186–2.738) | 0.006 | 2.283 (1.386–3.761) | 0.001 | |
| Ⅲ | 2.162 (1.297–3.603) | 0.003 | 2.474 (1.381–4.433) | 0.002 | |
| Ⅳ | 4.625 (1.978–10.815) | <0.001 | 4.780 (1.976–11.565) | 0.001 | |
| Adjuvant chemotherapy |  | 0.043 |  | 0.003 | |
| No | 1 |  | 1 |  | |
| Yes | 0.587 (0.478–0.988) |  | 0.567 (0.388–0.828) |  | |
| B3GNT3 |  | 0.025 |  | 0.008 | |
| Low | 1 |  | 1 |  | |
| High | 1.471 (1.049–2.064) |  | 1.665 (1.140–2.433) |  | |
| SLC3A2 |  | 0.002 |  | <0.001 | |
| Low | 1 |  | 1 |  | |
| High | 1.836 (1.248–2.701) |  | 3.439 (2.087–5.665) |  | |

AJCC, American Joint Committee on Cancer; CI, confidence interval; HR, hazard ratio.
