# Supplementary material for: Targeting N-glycosylation of 4F2hc mediated by glycosyltransferase B3GNT3 sensitizes ferroptosis of pancreatic ductal adenocarcinoma
Source: Cell Death Differ. 2023 Jul 21;30(8):1988–2004. doi: 10.1038/s41418-023-01188-z (PMC10406883; doi:10.1038/s41418-023-01188-z)
Supplement: Supplementary file 15 — Supplementary Figure and Table Legends [file 41418_2023_1188_MOESM15_ESM.docx]

**Supplementary Figure and Table Legends**

**Targeting *N*-glycosylation of 4F2hc** **mediated by glycosyltransferases B3GNT3** **sensitizes ferroptosis of pancreatic ductal adenocarcinoma**

Heng Ma^1^, Xianlong Chen^1^, Shengwei Mo^1^, Yue Zhang^1^, Xinxin Mao^1^, Jingci Chen^1^, Yilin Liu^1^, Wei-Min Tong^2^, Zhaohui Lu^1^, Shuangni Yu^1*^, Jie Chen^1^^*^

E-mail: ^1*^chenjie@pumch.cn; ^1*^yushn@pumch.cn

**Supplementary Figure 1.** ***N/O*-****glycoproteomic analyzes the proportion and function enrichment of differential glycoprotein.** **A–C.** Pie diagrams present the proportions of ferroptosis-related proteins (including 255 drivers, 208 suppressors, 125 Markers) **(A)**, differential glycopeptides **(B)**, and differential glycoproteins **(C)**. **D–F.** The most Gene Ontology (GO) terms for differential upregulated *N*- **(D)** and *O*-glycoproteins **(F). E–G.** Twenty important Kyoto Encyclopedia of Genes and Genomes (KEGG) pathways for differential upregulated *N*- **(E)** and *O*-glycoproteins **(G).** **H.** *N*-glycosylation sites of 4F2hc and B3GNT3 were predicted by NetNGlyc 1.0. **I.** The top 10 KEGG pathways enriched for upregulated genes in response to RSL3 treatment. **J.** Represent peptide of B3GNT3 was identified by LC–MS/MS. **K.** Coomassie blue stained SDS gels of affinity-purified protein complexes co-immunoprecipitated by the anti-B3GNT3 antibody from *B3GNT3*^OE^ PANC-1 cells (line 3). A representative peptide of 4F2hc was identified by LC–MS/MS. **L.** Co-IP analysis of the interaction between 4F2hc and B3GNT3 in PANC-1 cells treated with RSL3 or not.

**Supplementary Figure 2.** **Investigation of four glycosyltransferase genes and the expression of 4F2hc and B3GNT3.** **A–D.** The mRNA levels of indicated glycosyltransferase genes in tumor tissue and adjacent normal tissue were analyzed from Gene Expression Profiling Interactive Analysis (GEPIA). Box and whisker plots were used to present the data distribution, the median, 25th, and 75th percentiles were shown in boxes. The red plots denote the tumor and the gray plots denote normal tissue, “num” is the number of cases. **E–H.** Kaplan–Meier meta-analyses of overall survival (OS) stratified by the expression of indicated glycosyltransferase genes in PDAC patients using TCGA datasets. n is the number of samples. **I, J.** The mRNA levels of *SLC3A2* and *B3GNT3* in PDAC patients and normal controls were mined from the TCGA datasets (http://cancergenome.nih.gov/). Box and whisker plots were used to present the data distribution, and the median, 25^th^, and 75th percentiles are shown in boxes. T: tumor; N: adjacent normal tissue; num is the number of cases. **K.** TMA panorama of IHC staining for B3GNT3 and 4F2hc. **L.** The proportion of 4F2hc and B3GNT3 positive patients under different IHC scores. **M.** TMA panorama of mIHC/IF staining for 4F2hc, B3NGT3, xCT, NRF2, CK, and DAPI. **N.** Representative multiplex IHC images of the expression of low/high 4F2hc and low/high B3GNT3. Scale bar: 100 µm.

**Supplementary Figure 3.** **Functional analysis of 4F2hc and its *N*-glycosylation in PDAC cells.** **A.** Correlation analysis between CTRP drug sensitivity and indicated mRNA expression by Gene Set Cancer Analysis (http://bioinfo.life.hust.edu.cn/web/GSCALite/). **B****.** CTRP analysis of the response distribution of human cell lines with *SLC3A2* gene expression under RSL3 AUC (area under the curve). **C, D.** BxPC-3 cells were treated with TM in a dose- and time-dependent escalation manner and the expression change of 4F2hc and GPX4 were determined by western blotting. The purple and blue triangles represent high- and low- glycosylation, respectively. **E.** The mRNA and protein levels of 4F2hc in sh*SLC3A2* PANC-1 cells were determined by qRT-PCR and western blotting. **F.** The protein levels of 4F2hc, xCT, DHODH, and GPX4 in sh*SLC3A2* PANC-1 were analyzed by western blot. **G.** Schematic diagram of full-length structural domain of human 4F2hc and strategy to engineer mutants of N365Q and 4NQ (asparagine (N) mutated into glutamine (Q)) of 4F2hc. N365Q denotes asparagine (N) 365 mutated into glutamine (Q); 4NQ denotes asparagine 365, 381, 424, and 506 mutated into glutamine (Q). ****P* < 0.001.

**Supplementary Figure 4. Functional analysis of B3GNT3 and 4F2hc in PDAC cells.** **A.** CTRP analysis of the response distribution of human cell lines with *B3GNT3* gene expression under RSL3 AUC. **B.** The cell viability of *B3GNT3*^KO^ PANC-1 cells treated with the indicated compound. Treatment concentration: 0.8 μM RSL3, 2 μM liproxstain-1, 10 μM UAMC, 50 μM vitamin E, 50 μM Z-VAD-FMK, and 50 μM Necrostain-1. **C.** The mRNA levels of *B3GNT3* in PANC-1 and MIA PaCa-2 cells transfected with or without 1.25 μg overexpression *B3GNT3* plasmids. **D.** Cell viability analysis of the rescue effect of *SLC3A2*^OE^ and *B3GNT3*^OE^ in sh*SLC3A2* PANC-1 and MIA PaCa-2 cells treated with or without RSL3. **E.** Diagram of strategy to design the overexpression/deletion plasmids of enzyme active site of B3GNT3, respectively. 122–311^OE^ and 122–311^del^ denote overexpression and deletion of the glucosyltransferase active site of B3GNT3, respectively. **F,** **G.** CHX-chase analysis for PANC-1 and MIA PaCa-2 cells transfected with indicated plasmids. Cells were treated with 20 μM CHX at the indicated intervals, and then the protein expression of 4F2hc and GPX4 was measured by immunoblotting, and the intensity of 4F2hc and GPX4 in 122-311^OE^ and 122-311^del^ groups were quantified and normalized to the 0-time point of vehicle group (J). **P* < 0.05, ***P* < 0.01, ****P* < 0.001, *****P* < 0.0001, ns is no significant difference.

**Supplementary Figure 5.** **De-glycosylation of 4F2hc destabilized its interaction and membrane co-localization with xCT.** **A.** Sequence alignment of glycosylation site Asn365 on 4F2hc across different species. **B.** Western blotting analyzing the 4F2hc, xCT, B3GNT3, and GPX4 levels in sh*SLC3A2* PANC-1 cells transfected with or without 4NQ or N365Q. **C.** The protein levels of 4F2hc, NRF2, xCT, B3GNT3, and *β*-actin in PANC-1 cells treated with 0.8 μM RSL3 in the presence or absence of 10 µg/ml TM. **D.** Immunofluorescence image of PANC-1 cells treated with 0.8 μM RSL3, 10 µg/ml TM, or RSL3 + TM for 12 h. Scale bar, 20 μm. **E, F.** Co-IP analysis of PANC-1 cell lysates with anti-4F2hc (A) and anti-xCT (B) antibodies (Abcam#175186, 55kDa). The precipitated complexes were detected by western blotting with the indicated antibodies. IP, immunoprecipitation; IB, immunoblotting. **G, H.** The confocal image determines the membrane expression of 4F2hc and xCT in shControl PANC-1 cells (**G**), (Scale bar, 20 μm) and sh*SLC3A2* PANC-1 cells transfected with N365Q or 4NQ (**H**), (Scale bar, 25 μm).

**Supplementary Figure 6. Impairing the *N*-glycosylation process sensitizes PDAC cells to gemcitabine. A, B.** Treatment with RSL3, TM, or RSL3 plus TM enhanced the sensitivity of PANC-1 cells to gemcitabine. Cell viability was shown in the heat map **(A)** and statistical graph **(B)**. **C, D.** Knockdown of *SLC3A2* or combined with TM enhanced the sensitivity of PANC-1 cells to gemcitabine. Cell viability was shown in the heat map **(C)** and statistical graph **(D)**. All data are shown as mean ± SD. Statistical significance among indicated groups was assessed by ANOVA analysis. **P* < 0.05, ***P* < 0.01, *****P* < 0.0001, ns is no significant difference.

**Supplementary Figure 7. Knockout of *B3GNT3*** **or knockdown *SLC3A2* limited the proliferative capacity of PDAC cells in vitro.** **A.** Wound healing assays were performed in Cas9^control^ and *B3GNT3*^KO^ PANC-1 and MIA PaCa-2 cells for 48 h and wound closure relative to 0 h was quantified using ImageJ. Scale bar, 250 μm. **B.** Cell migration and invasion were examined by Transwell assays after CRISPR–Cas9-mediated *B3GNT3* knockout in PANC-1 and MIA PaCa-2 cells. Scale bar, 50 μm. **C.** Colony-forming assays in Cas9^control^ and *B3GNT3*^KO^ PANC-1 and MIA PaCa-2 cells for 2 weeks. Representative images and quantitation are shown. **D.** The wound healing assay was performed in shControl and sh*SLC3A2* PANC-1 and MIA PaCa-2 cells for 48 h and wound closure relative to 0 h was quantified using ImageJ. Scale bar, 250 μm. **E.** Cell migration and invasion were examined by Transwell assays in PANC-1 and MIA PaCa-2 cells with the indicated genotypes. Scale bar, 50 μm. **F.** Colony-forming assays in PANC-1 and MIA PaCa-2 cells with the indicated genotypes for 2 weeks.

**Supplementary Figure 8. Effect of suppressing the glycosyltransferase activity of B3GNT3 on clone formation, migration, and invasion of PDAC cells.** **A.** Colony-forming assays in *Cas9*^Control^, *B3GNT3*^KO^, and *B3GNT3*^KO^ + 122-311^del^ PANC-1 and MIA PaCa-2 cells for 7 days. Representative images and quantitation are shown. **B.** Wound scratch assay was performed to analyze the wound closure (48 h). Scale bar, 250 μm. **C.** Cell migration (up panel) and cell invasion were evaluated by transwell assay (48 h) (bottom panel) in *Cas9*^Control^, *B3GNT3*^KO^, and *B3GNT3*^KO^ + 122-311^del^ PANC-1 and MIA PaCa-2 cells. Scale bar, 50 μm. **D.** Mice body weight measurement of PANC-1 xenografts tumors with indicated genotype. **E.** Body weight measurement of PANC-1 cell line xenografts with the indicated treatments. Data are shown as mean ± SD. ***P* < 0.01, *****P* < 0.001.

## Supplementary Figure 9. The proposed model of how B3GNT3-mediated *N*-glycosylation of 4F2hc potentiates ferroptosis resistance in the PDAC context. Detailed information is described in the discussion.

## Supplementary Table 1. The Data sets of ferroptosis-related genes (FRGs) and glycosyltransferases genes.

**Supplementary Table 2.** The sequences of the primers for qRT-PCR were used in this study.

**Supplementary Table 3.** Association of clinicopathological features with B3GNT3 and 4F2hc expression.

**Supplementary Table 4.** Univariate analysis of indicated factors potentially associated with progression-free survival and disease-specific survival.

**Supplementary Table 5.** Multivariate analysis of indicated factors potentially associated with progression-free survival and disease-specific survival.
