# Supplementary material for: Targeting N-glycosylation of 4F2hc mediated by glycosyltransferase B3GNT3 sensitizes ferroptosis of pancreatic ductal adenocarcinoma
Source: Cell Death Differ. 2023 Jul 21;30(8):1988–2004. doi: 10.1038/s41418-023-01188-z (PMC10406883; doi:10.1038/s41418-023-01188-z)
Supplement: Supplementary file 16 — Supplementary Materials and Methods [file 41418_2023_1188_MOESM16_ESM.docx]

**Supplementary Materials and Methods**

**Targeting *N*-glycosylation of 4F2hc** **mediated by glycosyltransferases B3GNT3** **sensitizes ferroptosis of pancreatic ductal adenocarcinoma**

Heng Ma^1^, Xianlong Chen^1^, Shengwei Mo^1^, Yue Zhang^1^, Xinxin Mao^1^, Jingci Chen^1^, Yilin Liu^1^, Wei-Min Tong^2^, Zhaohui Lu^1^, Shuangni Yu^1*^, Jie Chen^1^^*^

E-mail: ^1*^chenjie@pumch.cn; ^1*^yushn@pumch.cn

## Immunohistochemistry (IHC) and evaluation of immunostaining

IHC staining was performed as previously described^[1]^. Briefly, TMA specimens were heated to 60 °C to melt the wax, then subjected to standard deparaffinization and hydration. Heat-mediated antigen retrieval was carried out in a pressure cooker with EDTA buffer (PH 9.0) or citrate buffer (PH 6.0) at 95 °C for 10 min. After slides were cooled to room temperature and tissue endogenous peroxidase was quenched, TMAs sections were blocked in goat serum (ZLI-9096) for 30 min at room temperature and then incubated with primary antibodies: 4F2hc (Cell Signaling Technology, 47213, 1:200; Massachusetts, USA), B3GNT3 (Proteintech, #18098-1-AP, 1:100; Chicago, USA), Ki67 (ZSGB-BIO, ZM-0166, 1:100; Beijing, China) at 4 °C overnight. The slides were washed three times with PBST and incubated with biotin-conjugated secondary reagents for 30 min at room temperature. All TMAs slides were automatically digitally scanned at 400× magnification using NanoZoomer S360 (Hamamatsu, Japan) for visualization. The semi-quantitative immunoreactive scores for 4F2hc, B3GNT3, and Ki67 were used to evaluate the immunostaining, and any discrepancies were resolved by a consensus pancreatic cancer diagnostic pathologist (J.C.). Quantification of Ki67 staining was performed by counting the positive cells in the five random fields (at 400 × magnification) relative to the total number of cells. 4F2hc was localized to the cell membrane and B3GNT3 showed cytoplasmic staining, and scoring was performed using the H-score method. The H-score method takes each staining intensity ( which was assigned from 0 to 3: negative (score 0), low (score 1), medium (score 2), and high (score 3)) and the percentage of positive cells into account, which were similar to the method used by Weimin Wang^[2]^ and Chia-Wei Li et al.^[3]^, respectively. The final score was calculated using the following formula: H-score = [1× (% cells 1+) + 2× (% cells 2+) + 3× (% cells 3+)]. The expression of 4F2hc and B3GNT3 was divided into low and high based on the median values, in the meantime, X-tile (Yale University, New Haven, CT, USA) also was used to determine the best cutoff values for comparative analysis.

## Western blot and Co-immunoprecipitation (Co-IP)

Western blotting for protein analysis was performed using a standard protocol as previously described^[4]^. Briefly, 6 paired tumor tissues with adjacent normal tissues and cell pellets were ground and lysed in radioimmunoprecipitation assay (RIPA) buffer (APPLYGEN, C1053+) containing 1% protease inhibitor and 1% phosphatase inhibitors for 30 min on ice. Cell lines were lysed directly. After centrifugation at 12,000 × g for 10 min at 4°C, the supernatant proteins were quantified using BCA assay (Thermo Scientific, Pierce^TM^ BCA protein assay Kit, 23225). Thirty micrograms of protein were mixed with 5 × loading buffer and denatured in 95°C water bath. Western blot analysis was performed using primary antibodies against 4F2hc rabbit mAb (Cell Signaling, 47213, 1:1000), 4F2hc mouse mAb (Proteintech, 66883-1-Ig, 1:1000), xCT (Cell Signaling, 12691, 1:1000), B3GNT3 (Proteintech,18098-1-AP, 1:1000), B3GNT3 for IP (Thermo fisher scientific, PA5-112092, 5-10 µL/mg lysate), GPX4 (Abcam, ab125066, 1:1000), β-actin (Sigma, a5441, 1:1000), GAPDH (Cell Signaling, 5174S, 1:1000), NRF2 (Abcam, ab62352, 1:1000), DHODH (Proteintech, 14877-1-AP, 1:1000), FSP1(Proteintech, 20886-1-AP, 1:500), and FLAG (Cell Signaling, 8146, 1:1000). Detection of GAPDH or actin served as control for equal loading.

Co-IP assays were performed using the Immunoprecipitation Kit (Invitrogen, 10007D) according to standard protocols. 1 × 10^7^ cells were transfected with the indicated plasmid or treated with indicated reagents for the indicated times. After treatment, the cells were collected and lysed in ice-cold IP lysis buffer (Thermo Scientific, 87788) with 1% protease inhibitor and 1% phosphatase inhibitor for 30 min on ice. After centrifugation at 12,000 × g for 10 min at 4°C, the supernatant was quantified using BCA assay. 20 μL of protein was used as the input for western blotting. The indicated antibody was diluted in 200 μL of Ab binding and washing buffer to magnetic beads, then incubated with rotation for 60 min at room temperature. After the Ab-conjugated magnetic beads were prepared, add equal amounts of antigen were added to gently resuspend the magnetic bead-Ab complex and incubated with rotation overnight to immunoprecipitate the target antigen. Then, the magnetic bead-Ab-Ag complex was boiled with 20 μL Elution Buffer and 10 μL of pre-mixed NuPAGE^TM^ LDS Sample Buffer and NuPAGE Sample Reducing Agent at 70 °C for 10 min, and loaded supernatant onto a gel and subjected to western blot analysis. All original western blots are shown in the Supplementary original data.

## CRISPR-Cas9-mediated stable knockout cells generation

CRISPR-mediated knockout lentiviral packaging plasmids containing *B3GNT3* sgRNAs and control sgRNA were purchased from Syngentech. The constructs were confirmed using DNA target sequencing. PANC-1, MIA PaCa-2 and BxPC-3 KO cell lines were generated by lentivirus transduction or transfected with pLV_U6-*B3GNT3*-sgRNA using Lipofectamine 3000 (Invitrogen). Puromycin selection was performed to select the stable knockout cells according to the manufacturer’s instructions. The target sequence of *B3GNT3* is GGATGAAGTATCTCCGGCAC, which was reported and identified in pancreatic cancer by Rohitesh Gupta et al..^[5]^

## Plasmid construction, stable cell lines generation, and small interfering RNAs

For human *SLC3A2* knockdown, *SLC3A2*-specific shRNA constructs were synthesized by Shanghai Genechem Co., Ltd. and subcloned into the GV344 lentiviral vector. Cells (1 × 10^5^ per well) were seeded in 6-well plates and transduced with recombinant lentivirus vectors at the indicated MOI with 10 μg/ml polybrene for 2 days. Then, the stable knockdown cells were selected using 2 μg/ml puromycin. The sh*SLC3A2* target sequences were as follows: sh*SLC3A2*-1: 5′-TGGGTCCAATTCACAAGAA-3′, shSLC3A2-2: 5′-AGAAGAATGGTCTGGTGAA-3′, sh*SLC3A2*-3:5′-CGTGTCATTCTGGACCTTA-3′, and nontargeting sequence：5′- TTCTCCGAACGTGTCACGT-3′. For *B3GNT3* overexpression, cDNA encoding *B3GNT3* was cloned into the pcDNA3.1, which was purchased from WZ Biosciences Inc. The target sequence of OE-*B3GNT3* was as follows: F: 5′-CGCAAATGGGCGGTAGGCGTG-3′; R: 3′-CCTCTACAAATGTGGTATGGC-5′. For *B3GNT3* knockdown, si*B3GNT3* and scrambled control were synthesized by RioboBio. Cells were transfected with Lipofectamine^TM^ 3000 (Invitrogen, L3000015) and harvested after 48 h. Target gene expression was identified by qRT-PCR and western blotting. The target sequence of si*B3GNT3* was as follows: si*B3GNT3*-1: 5′-CGCAGCACGTTCAGAACTT-3′, si*B3GNT3*-2: 5′-TGCACCGCTTCCTACCTTA -3′, si*B3GNT3*-3: 5′-GGATGATGACGTCTTTGCA-3′.

The cDNA fragment that encoded full-length human *B3GNT3* with 3 × Flag-tag (NCBI reference sequence: NM_014256.4, Flag-B3GNT3), Flag-*B3GNT3* amino acid (aa) 122-311 (122-311^OE^), Flag-*B3GNT3* aa 122-311 deleted mutant (122-311^del^) were cloned into the GV657 vector. Human full-length *SLC3A2* with Myc-tag (NCBI reference sequence: NM_002394.6) were subcloned into a GV658 vector. Using the human *SLC3A2*-WT-Flag plasmid as a template, *SLC3A2*-N365Q-Flag (Asparagine (Asn, N) residue was changed to glutamine (Gln, Q)) and *SLC3A2*-4NQ-Flag (N365Q, N424Q, N481Q, and N506Q) were developed. All of those plasmids were ordered from Shanghai Genechem Co., Ltd and were verified by DNA sequencing. Cells with indicated genotypes were transfected with 2.5 μg plasmids per sample using Lipofectamine 3000 transfection reagent.

## LC-MS/MS for glycoproteomics analysis

All samples were sent for glycoproteomics analysis based on liquid chromatography-mass spectroscopy/mass spectroscopy (LC-MS/MS) which was performed by Beijing Qinglian Biotech, Co., Ltd.

**In-gel digestion and HILIC for glycopeptide enrichment** Briefly, after Coomassie blue staining, the stained protein band was carefully cut into 0.5-1 mm^3^ and washed with 200 μl MS water for 10 min each time. The cut gel cubes were then destained in 50 mM ammonium bicarbonate (ABC) with 50% acetonitrile (ACN) and dehydrated with 100% ACN until the gel turned white. After dehydration, the gel cubes were washed again. ACN was dehydrated until the colloidal particles turned white, and the gel cubes were dried for 10 min to evaporate the remaining ACN. The gel cubes were treated with 200 μL of 10 mM DTT for 1h and alkylated with 55 mM Iodoacetamide (IAM) for 30 min in the dark. Gel spots were slightly centrifuged with 100 μL 0.01 μg/μL trypsin and incubated overnight at 37 °C. Next, the enzymatic digestion was collected by centrifugation at low speed, and the supernatant was extracted in 100 μL of 0.1% formic acid (FA). All supernatants were combined and centrifuged at 12,000 g for 5 min and then lyophilized. The powder was resuspended in 0.1% FA and slowly loaded onto a C18 desalting column. After being washed with 0.1% FA and 3% ACN and eluted with elution buffer (0.1%FA, 70%ACN), the peptide was redissolved to pass through the HILIC column, then washed with 80% ACN and eluted with 5% ACN to collect glycopeptide.

**LC-MS/MS analysis** Mobile phase A (99.9% water, 0.1% FA) and mobile phase B (99.9% acetonitrile, 0.1% FA) were used, and the elution gradient was used from 5% to 90% mobile phase B for 78 min to separate peptides. The separated peptides were analyzed using an Orbitrap Fusion mass spectrometer (Thermo Fisher Scientific). The full scan and the MSI were analyzed with a range from m/z 300 to 1400 at a resolution of 120,000 (at m/z 200). The automatic gain control (AGC) was set at 5 × 10^5^ and the maximum ion injection time (MIT) was 100 ms. The top 25 most intense ions were selected for fragmentation in higher-energy collisional dissociation (HCD) and analyzed by MS/MS, the AGC was set at 5 × 10^3^, the MIT was 35 ms, and the dynamic range was 15s to avoid the repeated sections of the same ion peaks.

**The identification and quantitation of peptide & protein** Resulting in mass spectrometry data were searched against the *Homo Sapiens* SP database using bionic search engines. The database search parameters were set as follows: two maximum missed cleavage sites permitted by trypsin digestion, and the minimum and maximum peptide lengths were 6 and 144 amino acids, respectively. Twenty ppm precursor ion mass tolerance and 20 ppm product ion mass tolerance. Carbamidomethyl (C, +57.022 Da) was used for static modification. Oxidation modification (+15.995 Da) and acetylation were specified as dynamic modifications. The FDR for peptide and protein identification was set to 0.01.

## Immunofluorescence (IF) and confocal

Briefly, 1 × 10^4^ cells were seeded on glass coverslips (YA0350, Solarbio) in 24-well plates overnight. The cells were then treated with various compounds for the indicated times. After treatment, cells were fixed in 4% formaldehyde for 15 min and permeabilized with 1% Triton X-100 for 15 min. After blocking with 5% bovine serum albumin containing 0.1% Triton X-100 for 30 min at room temperature, cells were incubated with primary antibodies against 4F2hc mouse mAb (Proteintech, 66883-1-Ig, 1:100), xCT rabbit PolyAb (Cell Signaling, 12691, 1:50) for overnight. The coverslips were then washed with PBS and incubated with secondary antibodies conjugated with Alexa Fluor^®^ 488 (ZSGB-BIO, ZF-0512, 1:100) or Alexa Fluor^®^ 594 (ZSGB-BIO, ZF-0516, 1:100) for 1h at room temperature, and mounted with antifading mounting medium with DAPI. Fluorescent images of the cells were captured using a laser-scanning confocal microscope (Nikon, Japan).

## qRT-PCR

Total RNA from treated cells was extracted using the RNA-Quick Purification Kit (ES Science, RN001) and reverse transcribed to cDNA using the PrimeScript^TM^ RT reagent kit (TaKaRa, RR037A). qRT-PCR was performed using the SYBR Select Master Mix (ThermoFisher Scientific, 4472908). The expression of all genes was normalized to that of the housekeeping gene encoding GAPDH. Primer sequences were listed in **Supplementary Table 2**.

## Cell viability assay

Cell viability was measured using a CCK8 kit (Dojindo, CK04) as previously described. Briefly, 2 × 10^4^ cells/per well were seeded into 96-well plates. After cell attachment and growth for 12 h, cells were treated with indicated chemicals (drug concentrations are indicated for RSL3 or Erastin in dose/response curves unless otherwise stated) for the indicated time. Subsequently, 10 μL CCK-8 reagent and fresh medium were combined to make up 100 μL reaction volume per well for 1 hour at 37 °C, 5% CO_2_ in an incubator. The absorbance at 450 nm was determined using an EPOCH2T microplate reader (BioTek Instruments).

## Glutamate and GSH level measurement

Relative extracellular glutamate levels were determined using the Glutamine/Glutamate Determination Kit (Sigma-Aldrich, GLN1). Briefly, 500 µL of the extracellular medium was added into 1 mL Tris-EDTA-hydrazine buffer, 100 µL NAD solution, 10 µL ADP solution, and water to make up 2 mL of total reaction volume. Test samples were mixed by inversion, and the background reading was detected at 340 nm. Next, 20 µL of L-GLDH was added and incubated for 40 min at room temperature. The absorbance was read at 340 nm and the concentration of L-glutamate was calculated from standards curves and the dilution factors were multiplied to obtain the preliminary glutamate level.

GSH was detected using the GSH/GSSG Ratio Detection Assay Kit (Abcam, ab138881) according to the standard guidelines. Briefly, take one percent of 5 × 10^6^ cells lysed in 100 µL pre-cooled 1× Mammalian Lysis Buffer. The cells were homogenized quickly and then centrifuged at 15,000 × g for 15 min at 4 °C to collect the supernatant for the GSH assay. Enzymes in the samples were removed using Deproteinizing Sample Kit (Abcam, ab204708) and neutralized with NaHCO_3_. Then, 50 µL of GSH Assay Mixture was added to the GSH standard and test samples per well. The plate was incubated for 30 min at room temperature, and the resulting fluorescence was measured at Ex/Em = 490/520 nm using Synergy Mx microplate reader (BioTek). The amount of GSH was determined using a GSH standard curve and the concentration in each sample was corrected by multiplying the dilution factor.

## RNA sequencing

The RNA-seq was performed by Beijing Qinglian Biotech, Co., Ltd. Sequencing libraries were constructed according to the following steps. Briefly, the mRNA was purified using poly-T oligo-attached magnetic beads. Fragmentation of mRNA was carried out at elevated temperatures using fragmentation buffer and the first-strand cDNA was synthesized using random primers and oligonucleotides. Then, the Super Script II. Second strand cDNA was synthesized using dNTPs, DNA polymerase I, and RNase H. After purification using the AMPure XP system (Beckman Coulter, Beverly, CA, USA), the DNA fragments were selectively enriched through a 15-cycle PCR reaction to generate the final RNA-seq library. The products were subjected to purification and quality control and the sequencing library was run on the NovaSeq 6000 platform (Illumina). To obtain high-quality sequence clean data, Cutadapt v1.15 software was used to filter the sequencing data and was aligned to the reference genome using HISAT2 v20.5. The mapped reads were assembled using the StringTie software (<http://ccb.jhu.edu/software/stringtie/>). All the expressions of each gene were compared by HTSeq statistics and standardized using FPKM. Then all differentially expressed genes (DEGs) were analyzed using DESeq2. The screened conditions were set as follows: 1) expression difference multiple |log2FoldChange| > 1, 2) significant Q-value ≤ 0.05. The R language Pheatmap (1.0.8) software package was used to perform a clustering analysis of the DEGs in each group. Additionally, the Gene Ontology and Kyoto Encyclopedia of Genes and Genomes (KEGG) (P-value<0.05 was used as the threshold) were used to analyze the enrichment of DEGs in each group.

## Multiplex immunohistochemistry/immunofluorescence (mIHC/IF)

Briefly, TMA specimens were baked at 65 °C for 60 min and subjected to standard dewax and rehydration (through a graded series of ethanol solutions: 100% 1×10 min; 95% 1×10 min; and rinse in 70%). The slides were then placed in a microwave with AR6 antigen retrieval buffer for 1 min at high power and for an additional 15 min at low power. After covering slides with blocking buffer for 10 min at room temperature, TMAs were labeled with the primary antibody against CK for the first round of staining, followed by incubating slides with secondary antibodies (Polymer HRP Ms + Rb) for 10 min at room temperature, then Opal Fluorophore Working Solution (containing Opal 690 tyramide signal amplification reagent) was used to generate Opal signal. Subsequently, the primary-secondary-HRP complex was stripped for allowing the introduction of the next primary antibody against 4F2hc (Opal 520), B3GNT3 (Opal 570), SLC7A11 (Opal 620), and NRF2 (Opal 480), sequentially. Finally, DAPI working solution was applied for 5 min in a humidity chamber and covered with a mounting medium, then single-channel and merged images were acquired and analyzed using the Vectra Polaris multispectral slide scanner (Akoya Biosciences) and inForm software (V.2.4.2; PerkinElmer, Inc).

## Wound healing, transwell assay and colony formation

**Wound healing:** 1 × 10^5^ indicated cells were seeded onto 6-well plates and cultured overnight until they reached more than 90% confluence. Then, a 200 μL size pipette tip was used to scratch the cell layer and washed with PBS to remove the detached cells three times. The same positions in each scratched field were photographed at 0, 12, 24, and 48 h, respectively. Image J software was used to quantify the width of the cell-covered area.

**Transwell assays** were performed to analyze cell migration and invasion. The indicated cells were suspended in serum-free medium and transferred to Matrigel-uncoated (for migration) and Matrigel-coated (for invasion) upper chambers and a medium containing 20% serum was added to the lower chamber. After incubation for the indicated times, cells were fixed in 4% paraformaldehyde and stained with 1% crystal violet. The number of migrated and invaded cells was counted in five random fields.

**Colony formation:** Approximately 800 cells were seeded into 6-well plates and transfected with indicated plasmids in a cell incubator for 1 week (culture medium was changed per 3 days). Then cells were fixed in 4% paraformaldehyde for 15 min and stained with 1% crystal violet for 30 min. The cell colonies were photographed and quantified.

**References**

1. Chen X, Mo S, Zhang Y, Ma H, Lu Z, Yu S, et al. Analysis of a novel immune checkpoint, Siglec-15, in pancreatic ductal adenocarcinoma. J Pathol Clin Res. 2022;8:268-278.

2. Wang W, Green M, Choi JE, Gijon M, Kennedy PD, Johnson JK, et al. CD8(+) T cells regulate tumour ferroptosis during cancer immunotherapy. Nature. 2019;569:270-274.

3. Li CW, Lim SO, Chung EM, Kim YS, Park AH, Yao J, et al. Eradication of Triple-Negative Breast Cancer Cells by Targeting Glycosylated PD-L1. Cancer Cell. 2018;33:187-201 e10.

4. Wang N, Ma H, Li J, Meng C, Zou J, Wang H, et al. HSF1 functions as a key defender against palmitic acid-induced ferroptosis in cardiomyocytes. J Mol Cell Cardiol. 2021;150:65-76.

5. Gupta R, Leon F, Thompson CM, Nimmakayala R, Karmakar S, Nallasamy P, et al. Global analysis of human glycosyltransferases reveals novel targets for pancreatic cancer pathogenesis. Br J Cancer. 2020;122:1661-1672.
