# Supplementary material for: Targeting N-glycosylation of 4F2hc mediated by glycosyltransferase B3GNT3 sensitizes ferroptosis of pancreatic ductal adenocarcinoma
Source: Cell Death Differ. 2023 Jul 21;30(8):1988–2004. doi: 10.1038/s41418-023-01188-z (PMC10406883; doi:10.1038/s41418-023-01188-z)

**Supplementary File**

**Uncropped original western blots**

**Targeting *N*-glycosylation of 4F2hc** **mediated by glycosyltransferases B3GNT3** **sensitizes ferroptosis of pancreatic ductal adenocarcinoma**

Heng Ma^1^, Xianlong Chen^1^, Shengwei Mo^1^, Yue Zhang^1^, Xinxin Mao^1^, Jingci Chen^1^, Yilin Liu^1^, Wei-Min Tong^2^, Zhaohui Lu^1^, Shuangni Yu^1*^, Jie Chen^1^^*^

^1^Department of Pathology, Peking Union Medical College Hospital, Peking Union Medical College and Chinese Academy of Medical Science, Beijing, 100730, China

^2^Department of Pathology, Institute of Basic Medical Sciences, Peking Union Medical College and Chinese Academy of Medical Science, Beijing, 100730, China.

**^*^Corresponding Author:** Jie Chen, Department of Pathology, Peking Union Medical College Hospital, Peking Union Medical College and Chinese Academy of Medical Science, Beijing, 100730, China. E-mail: chenjie@pumch.cn

Shuangni Yu, Department of Pathology, Peking Union Medical College Hospital, Peking Union Medical College and Chinese Academy of Medical Science, Beijing, 100730, China. E-mail: [yushn@pumch.cn](mailto:yushn@pumch.cn)


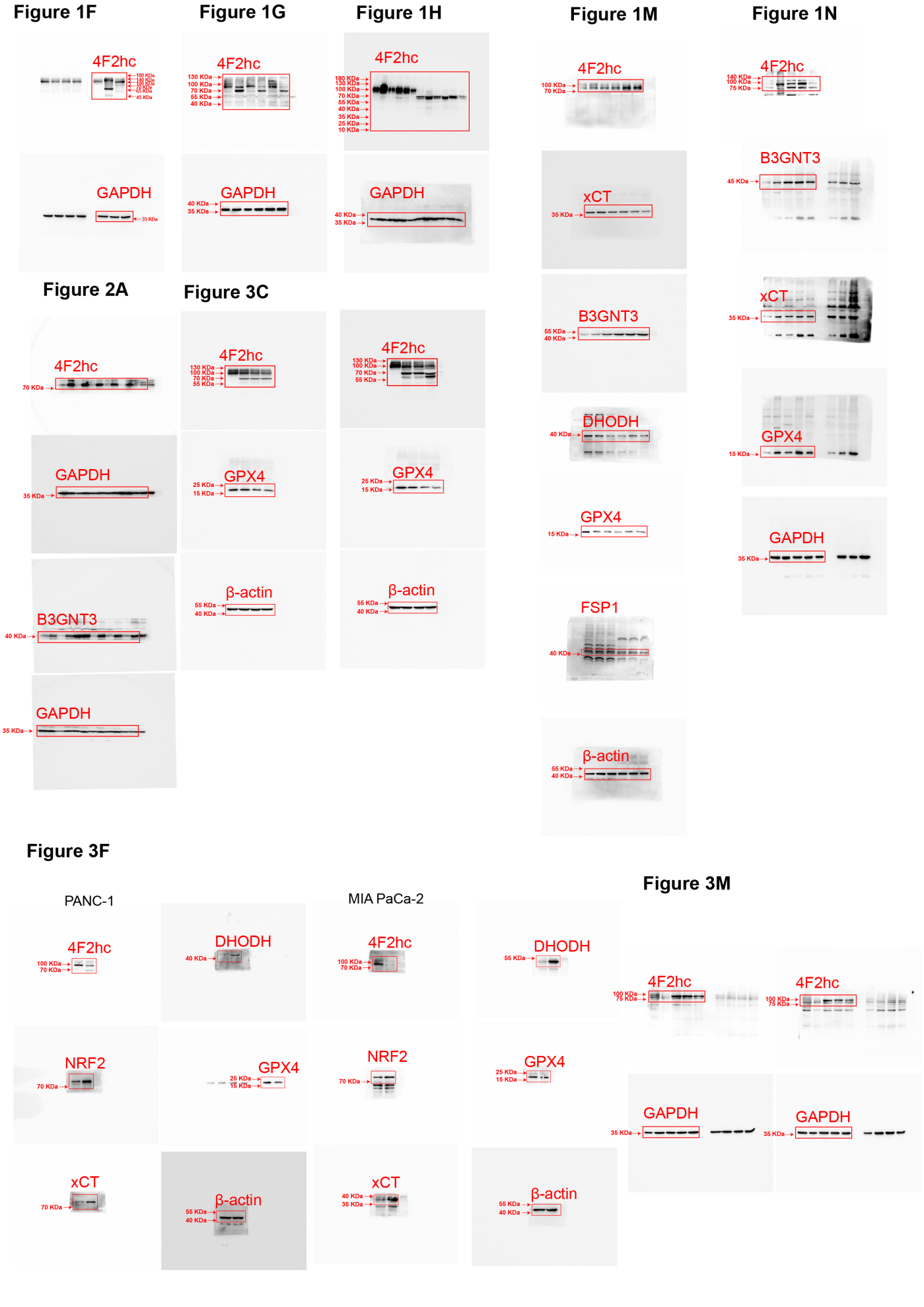


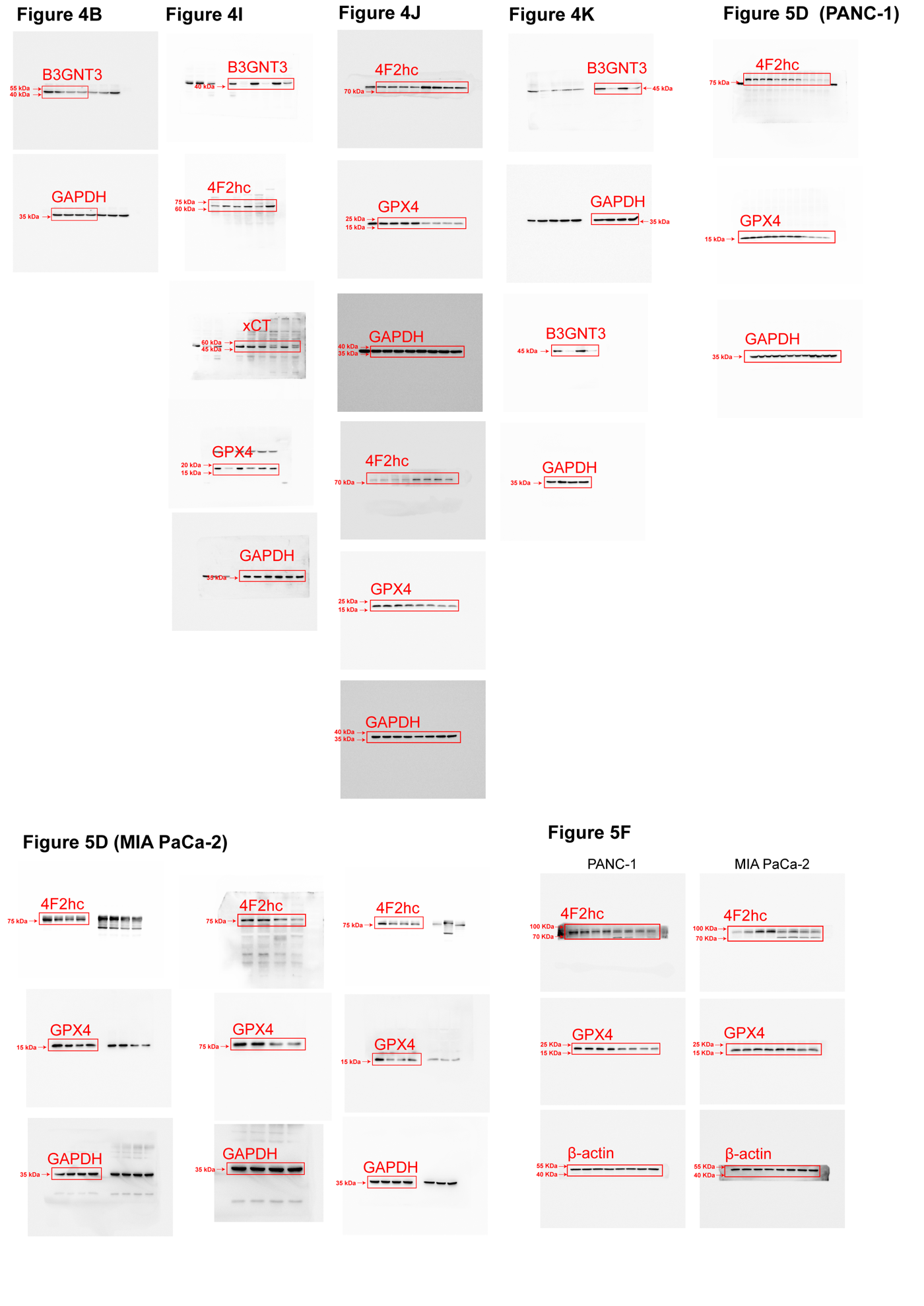

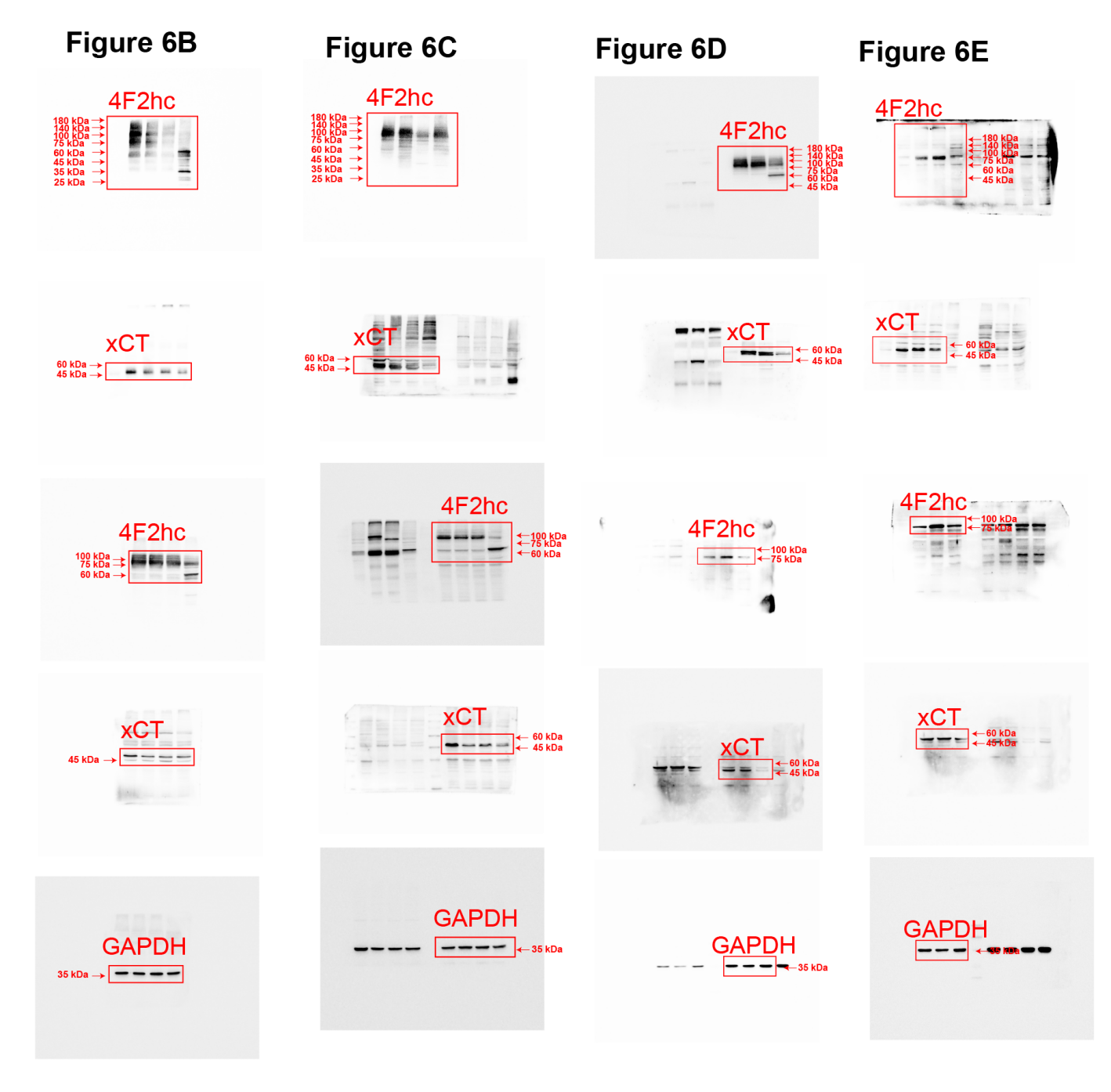

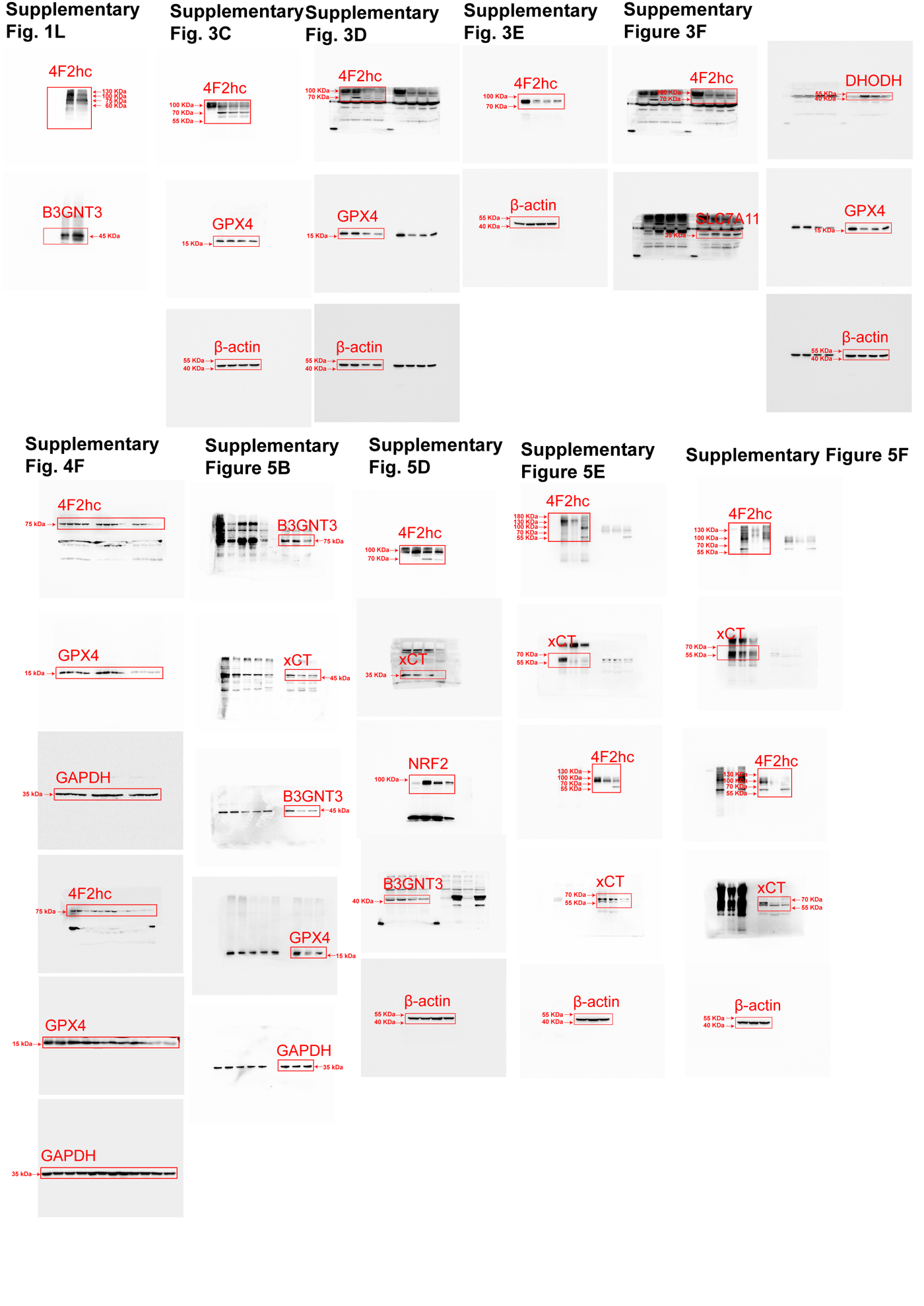

Supplement: Supplementary file 17 — Original File of Western Bolts [file 41418_2023_1188_MOESM17_ESM.docx]
